# Sex Hormone Profiles in Patients With Torsades de Pointes Ventricular Tachycardia: A Clinical-Electrophysiological Translational Study

**DOI:** 10.1016/j.jacep.2025.07.009

**Published:** 2025-08-28

**Authors:** Pietro Enea Lazzerini, Vamsi Krishna Murthy Ginjupalli, Jean-Baptiste Reisqs, Iacopo Bertolozzi, Silvia Cantara, Maria Grazia Castagna, Riccardo Accioli, Antonio D’Errico, Alessandra Cartocci, Anna Cantore, Viola Salvini, Decoroso Verrengia, Fabio Salvadori, Tommaso Marzotti, Matteo Capecchi, Stefania Bisogno, Michele Voglino, Sciaila Bernardini, Yongxia Sarah Qu, Franco Laghi-Pasini, Maurizio Acampa, Antonio Abbate, Pier Leopoldo Capecchi, Mohamed Boutjdir

**Affiliations:** a Department of Medical Sciences, Surgery and Neurosciences, Division of Internal Medicine and Geriatrics, Electroimmunology Unit, University of Siena, Siena, Italy;; b Cardiovascular Research Program, Veterans Affairs New York Harbor Healthcare System, State University of New York Downstate, New York, New York, USA;; c Cardiology Intensive Therapy Unit, Department of Internal Medicine, Nuovo Ospedale San Giovanni di Dio, Florence, Italy;; d Laboratory of Clinical and Translational Research, University Hospital of Siena, Siena, Italy;; e Department of Medical Biotechnologies, University of Siena, Siena, Italy;; f Department of Cardiology, New York Presbyterian Brooklyn Methodist Hospital, Brooklyn, New York, USA;; g Stroke Unit, University Hospital of Siena, Siena, Italy;; h Division of Cardiology, and Berne Cardiovascular Research Center, School of Medicine, University of Virginia, Charlottesville, Virginia, USA;; i New York University Grossman School of Medicine, New York, New York, USA.

**Keywords:** action potential duration, hormonal therapies, sex hormones, torsades de pointes, ventricular repolarization

## Abstract

**BACKGROUND:**

Female sex is a well-recognized risk factor for long QT syndrome and torsades de pointes (TdP), likely reflecting the influence of sex hormones on ventricular repolarization. Overall, estradiol prolongs, whereas progesterone and testosterone shorten, heart rate-corrected QT interval. However, no studies have comprehensively evaluated sex hormone levels in male and female long QT syndrome patients developing TdP, nor their implications in terms of clinical outcomes and electrophysiological changes.

**OBJECTIVES:**

This study was aimed at determining the sex hormones profiles in male and female TdP patients, and defining their role in this clinical setting.

**METHODS:**

The authors investigated: 1) the levels of sex hormones in a prospective cohort of male and female patients who developed TdP; 2) the relationship between sex hormones and arrhythmia outcome in TdP men and women; 3) the in vitro impact of sex-specific TdP hormone profiles on guinea pig ventricular myocyte and human-induced pluripotent stem cell–derived cardiomyocyte action potential duration, and their modulation by sex-specific hormonal interventions.

**RESULTS:**

Over 13 years, 68 TdP patients (42 female) were consecutively enrolled. Compared to control subjects, a differential sex hormone profile was observed in TdP men and women, primarily reduced testosterone in male patients and increased 17β-estradiol in female patients. Within the TdP cohort, lower testosterone in men and higher 17β-estradiol in women were associated with a worse short-term arrhythmia outcome. In vitro reproduction of sex-specific TdP hormone profiles prolonged action potential duration in sex-matched cardiomyocytes, an effect reversed by the addition of testosterone in male patients and progesterone in female patients, respectively.

**CONCLUSIONS:**

Different sex hormone profiles, primarily low testosterone in male patients and high 17β-estradiol in female patients, are associated with TdP occurrence and outcome in men and women. These endocrine milieus act, at least in part, via direct and reversible effects on cardiac electrophysiology, thereby supporting the antiarrhythmic potential of sex-specific hormonal-modulating therapies.

## CENTRAL ILLUSTRATION

APD = action potential duration; TdP = torsades de pointes.

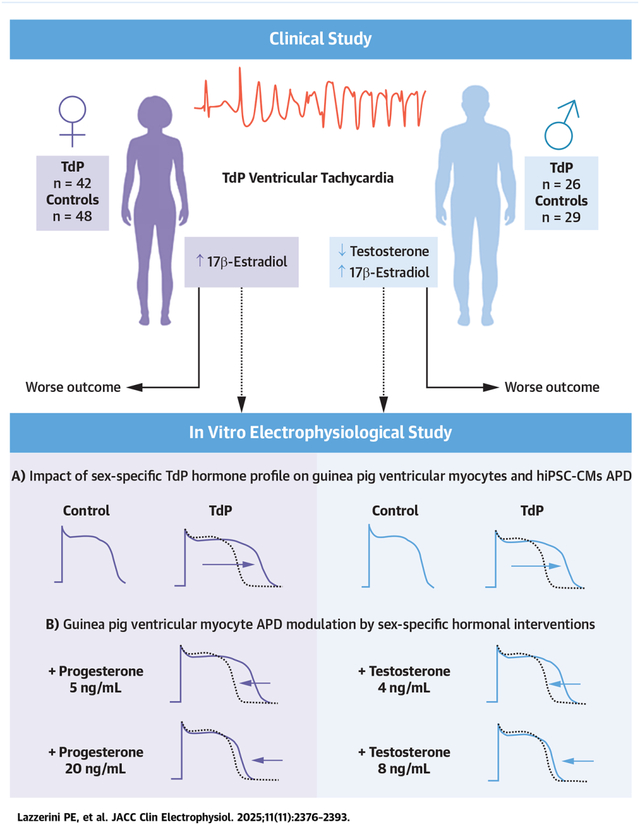

Female sex is a well-recognized risk factor for long QT syndrome (LQTS) and torsades de pointes (TdP) ventricular tachycardia.^1^ Women normally have longer heart rate-corrected QT interval, or QTc,^1^ and are at significantly greater risk of developing drug-induced TdP, than men are.^2^ Female sex is also independently associated with an increased incidence of syncope and sudden cardiac death (SCD) in the congenital LQTS.^1^ Thus, it is widely accepted that the cutoff values for QTc prolongation are sex-related.^2^

The basis for sex difference in LQTS/TdP risk is unclear, but is likely to reflect the influence of sex hormones on ventricular repolarization. Sex differences in QTc interval start with puberty, then gradually decrease with age, due to QTc lengthening in male patients until reaching the level of women around the age of female menopause; this strongly implicates the sex hormones in the differences between men and women in ventricular repolarization.^3^ Overall, estradiol is considered to promote QTc lengthening, whereas progesterone and testosterone promote QTc shortening.^4^ In line with this premise, the shorter QT interval of virilized, compared with normal, women and the longer QT interval of orchiectomized, compared to normal men, argue for a role for testosterone in shortening male ventricular repolarization.^5^ Moreover, women of childbearing age are at greater risk of QT prolongation during the follicular phase (high estradiol and low progesterone levels),^6^ whereas LQTS carriers have a reduced risk for cardiac events during pregnancy (high progesterone levels).^7^

The basic mechanisms underlying the impact of sex hormones on ventricular repolarization are not completely defined, but increasing evidence points to the direct modulating effects on several ion currents critically involved in determining action potential duration (APD) and hence QT interval duration. Preclinical studies suggest that testosterone can shorten QTc by both increasing the repolarizing potassium currents I_Kr_ (rapid delayed rectifier potassium current) and I_Ks_ (slow delayed rectifier potassium current) and decreasing the depolarizing L-type calcium current (I_CaL_).^4^ Similar effects on I_Ks_ (increase) and I_CaL_ (decrease) were also demonstrated for progesterone.^4^ Conversely, QTc-prolonging effects of estradiol seem to be principally mediated by a strong inhibition of the I_Kr_ current, only in part counteracted by concomitant effects on I_Ks_ (increase) and I_CaL_ (decrease).^4^ The clinical relevance of these activities is supported by preliminary data from small randomized controlled trials suggesting the effectiveness of transdermal testosterone and oral progesterone in attenuating drug-induced QTc lengthening in men and women, respectively.^8–10^

To date no studies have specifically and comprehensively evaluated the profiles of sex hormones levels, stratified by sex, in patients with LQTS who actually developed TdP. To our knowledge, the only available study is a report involving a small cohort of 7 male patients with TdP, in whom Salem et al^11^ assessed levels of testosterone and gonadotropins. In all these patients, hypogonadism was diagnosed, and after correction of low testosterone levels, QTc shortened, and TdP did not reoccur. Conversely, no information currently exists regarding the hormonal profile in women with TdP. The relevance of this subject is further strengthened by the evidence that the Oregon SUDS (Sudden Unexpected Death Study) has found that estrogen levels are higher in both male and female victims of SCD, whereas higher testosterone levels are associated with lower rates of SCD in men.^12^ However, this community-based study did not investigate the clinical and mechanistic bases underlying this association.^12^

The overall hypothesis is that sex-specific hormone imbalances (ie, low testosterone in men and high estradiol and/or low progesterone in women) can differentially promote LQTS and TdP in male patients and female patients via direct effects on cardiac electrophysiology. Thus, this study was aimed at determining the sex hormones profiles in male and female TdP patients, and define their role in this clinical setting. To address this objective, we investigated the following: 1) the levels of sex hormones in a cohort of men and women who presented with extreme QTc prolongation complicated with TdP, consecutively collected from the general population over 13 years; 2) the relationship between sex hormones and arrhythmia outcome in TdP male patients and female patients; 3) the in vitro impact of sex-specific TdP hormone profiles on guinea pig ventricular myocyte and human-induced pluripotent stem cell–derived cardiomyocytes (hiPSC-CMs) APD and its modulation by sex-specific hormonal interventions.

## METHODS

### STUDY POPULATIONS.

Local Ethical Committee (Comitato Etico Regionale per la Sperimentazione Clinica della Regione Toscana, Sezione Area Vasta Sud Est) approved the study, and patients gave their oral and written informed consent in accordance with the principles of the Declaration of Helsinki.

Since 2008, we have been prospectively enrolling consecutive patients who developed TdP, independent of ongoing therapies and concomitant diseases. On December 31, 2021, the cohort consisted of 68 patients, including 42 female patients and 26 male patients. Demographic, clinical, and laboratory characteristics of study patients, as well as ongoing treatment with QTc prolonging medications are provided in Table 1.

As a control, a group of 77 subjects (48 female and 29 male) comparable to TdP patients, but without LQTS/TdP, was additionally enrolled (demography and clinical characteristics depicted in Supplemental Table 1.

### SEX HORMONES MEASUREMENT.

In all subjects under study, a blood sample was obtained to measure circulating sex hormones. Specifically, in patients with TdP, the blood withdrawal was performed within 24 hours from arrhythmia occurrence. The levels of the following sex hormones were assessed: testosterone (total and free), SHBG (a sex-hormone binding globulin), androstenedione, 17β-estradiol, progesterone, follicle stimulating hormone (FSH), and luteinizing hormone (LH). All measurements were performed by an automatic chemiluminescent immunoassay system. More details are provided in the Supplemental Methods.

### ELECTROCARDIOGRAM RECORDINGS.

Diagnosis of TdP was based on the presence of at least 1 episode of polymorphic ventricular tachycardia and a rate ranging from 160 to 240 beats/min, associated with QTc prolongation.^2^ The details of QTc measurement are reported in the Supplemental Methods. TdP was defined as “complicated” when it degenerated into ventricular fibrillation (VF)/sudden cardiac arrest (SCA), and/or required electric shock.

### ACTION POTENTIAL RECORDINGS FROM GUINEA PIG SINGLE VENTRICULAR MYOCYTE AND HUMAN-INDUCED PLURIPOTENT STEM CELL–DERIVED CARDIOMYOCYTES (HIPSC-CMS).

Ventricular myocytes from guinea pig were isolated as previously reported,^13–15^ and more details are described in the Supplemental Methods. hiPSC–CMs were obtained as detailed in the Supplemental Methods. Action potentials were recorded from single ventricular myocytes or hiPSC-CMs before and after treatment with sex hormones in the whole-cell current-clamp configuration of the patch-clamp technique using an Axopatch-200B amplifier (Axon Instruments, Inc) by passing depolarizing currents at subthreshold intensity. APD at 90% full repolarization (APD_90_) was measured by Clampfit and analyzed using PRISM10 software (GraphPad). More details of the experimental protocol are provided in the Supplemental Methods and in Supplemental Figures 1 and 2.

### STATISTICAL ANALYSIS.

We performed power analyses stratified by sex, based on the well-established epidemiological evidence that risk for TdP is remarkably greater in women than in men, by a factor of approximately 2-fold.^2^

The sample size of the TdP and control groups were estimated independently for male and female subjects, by focusing on the first objective and using the 2-sided Mann-Whitney *U* test. An α of 0.05 and a large effect size of 0.80 were considered for male and female subjects. Thus, we estimated a sample size of 27 male subjects per group, setting a power of 0.80, and a sample size of 44 female subjects per group, setting the power at 0.95.

Based on data distribution, the following parametric and nonparametric statistical analyses were carried out: the 2-tailed unpaired Student’s *t*-test, or the 2-tailed Mann-Whitney *U* test to evaluate differences in quantitative variables between 2 groups of data (comparisons of age and sex hormones in TdP patients vs control subjects, and in patients with complicated vs uncomplicated TdP); the Spearman coefficient to verify possible statistical correlation between quantitative variables in patients with TdP (sex hormones); the 2-sided Fisher exact test, was performed to evaluate statistical association between categorical variables (comparisons of frequencies of sex hormones alterations and comorbidities in TdP patients vs control subjects, and sex hormones alterations in patients with complicated vs uncomplicated TdP). Moreover, the Kruskal-Wallis test and the Dunn test as post hoc analysis were performed to evaluate differences in quantitative variables (testosterone and 17-β estradiol) among 4 groups: male TdP patients with peripheral, central, and mixed hypogonadism and control subjects. The 17-β estradiol assay had a detection limit of 20 pg/mL. Statistical handling of values <20 pg/mL is detailed in the Supplemental Methods. The impact of different treatments on APD_90_ in vitro were statistically analyzed by the repeated measures analysis of variance, and post hoc multiple paired *t*-test with false discovery rate correction.

Values of *P* ≤ 0.05 were considered significant (GraphPad-InStat, version 3.06 for Windows 2000).

## RESULTS

### TdP PATIENTS’ CHARACTERISTICS.

As expected by the epidemiology of the arrhythmia, patients who developed TdP were more commonly women (62%), elderly (median 81 years), and presented with a high burden of QT-prolonging risk factors of acquired origin (on average 4.9 factors per patient), including those “conventional,” primarily cardiac diseases (82%), electrolyte imbalances (71%), and QT-prolonging drugs (61%), and “nonconventional.” such as systemic inflammation (81%)^16,17^ and anti-Ro/Sjogren syndrome-related antigen A autoantibodies (55%)^18–20^ (Table 1). Six patients aged <60 years underwent genetic testing for inherited LQTS, and 2 of them presented with pathogenic or likely pathogenic variants. Specifically, they include a 49-year-old man with no family history of LQTS/SCD who tested positive for a pathogenic variant on *SCN5A*, and a 59-year-old woman with a family history of LQTS and SCD who showed a likely pathogenic variant on *KCNH2*.

Nine TdP male patients were under current treatment with drugs potentially influencing circulating levels of sex hormones, including 6 with androgen-deprivation therapy and 2 with opioids. Specifically, 3 were treated with gonadotrophin-releasing hormone-receptor agonists/antagonists (2 leuprolide, 1 triptorelin), 2 with nonsteroidal androgen-receptor antagonists (bicalutamide), 2 with methadone maintenance therapy, 1 with morphine/codeine chronic analgesic therapy, and 1 with the 5-α reductase inhibitor dutasteride. Only 1 of the 42 TdP female patients was being treated with drugs potentially affecting sex hormones when TdP occurred (ie, the opioid fentanyl as chronic analgesic therapy), whereas none were under hormone replacement therapy.

### SEX HORMONES LEVELS IN MALE SUBJECTS.

**FIGURE 1:**
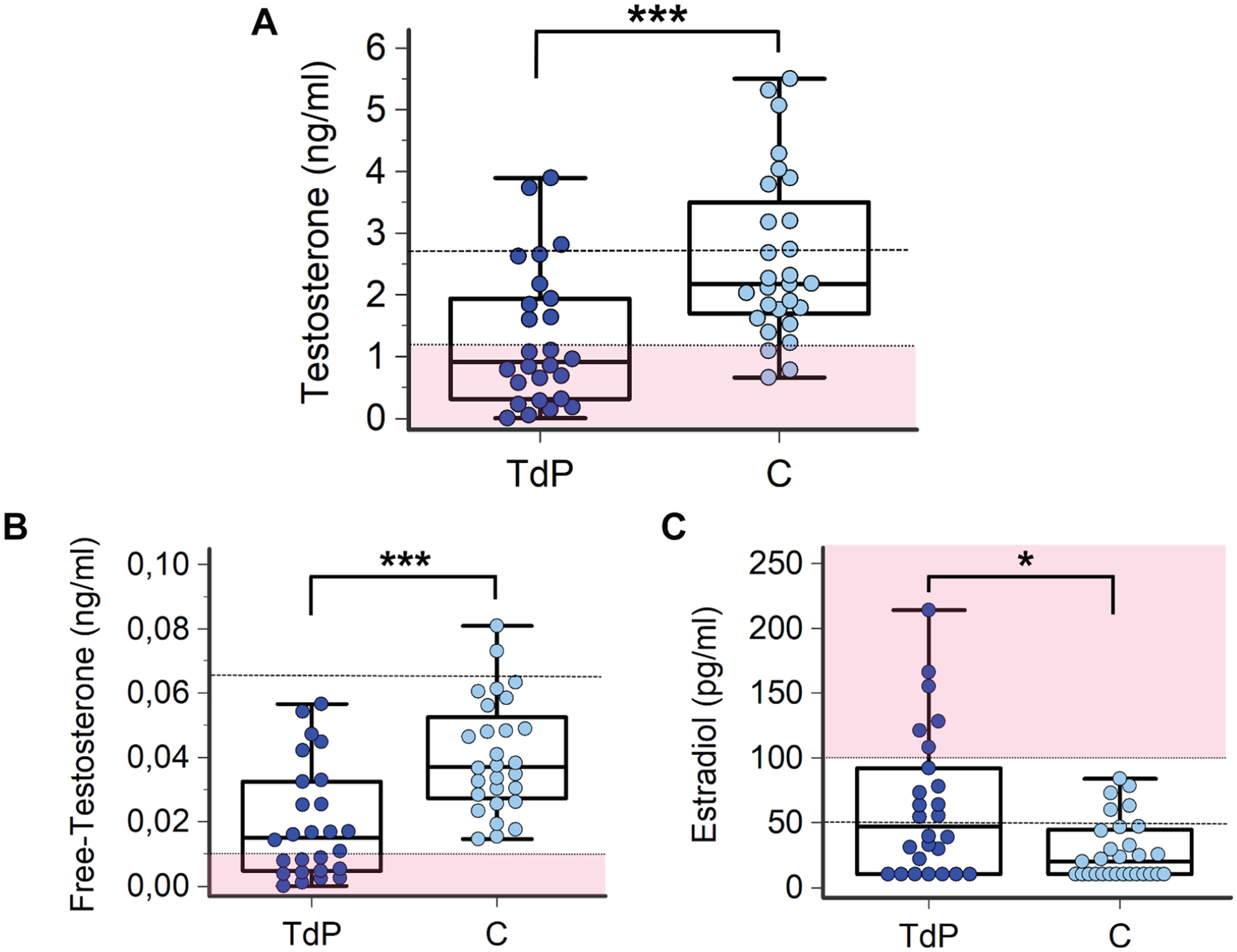
Circulating Testosterone and 17-β Estradiol Levels in Male Patients With TdP and Control Subjects Torsades de pointes (TdP) patients, n = 26; control subjects (C), n = 29. (A) Total testosterone: horizontal black dotted line indicates the lower limit of reference values in men (ie, 2.7 ng/mL); horizontal dotted line with pink below indicates the upper limit of reference values in premenopausal women (ie, 1.1 ng/mL). (B) Free testosterone: horizontal black dotted line indicates the lower limit of reference values in men (ie, 0.065 ng/mL); horizontal dotted line with pink below indicates the upper limit of reference values in premenopausal women (ie, 0.01 ng/mL). (C) 17-β estradiol: horizontal black dotted line indicates the upper limit of reference values in men (ie, 50 pg/mL); horizontal dotted line with pink above indicates values included in a premenopausal female range (ie, $100 pg/mL). Two-tailed Mann-Whitney *U* test: *P < 0.05, ***P < 0.001.

When compared to comorbidity control subjects, male TdP patients showed significantly lower total testosterone levels (over 50% reduction, with mean levels distinctly below the lower reference limit), and a higher prevalence of hypogonadism, especially profound hypogonadism with premenopausal female range levels (≤1.1 ng/mL; 62% vs 10%; *P* < 0.001) (Table 2, Figure 1). Such a difference was emphasized when free-testosterone levels were considered, because very low concentrations (≤0.01 ng/mL) were found in 42% of TdP patients vs none of the control subjects (Table 2, Figure 1). Moreover, in male patients with TdP, circulating 17-β estradiol was significantly higher (~2×) than in control subjects, with mean levels above the upper normal limit for men. In almost one-quarter of cases (23%), 17-β estradiol concentrations were found to be very high–in a range usually observed in premenopausal women (≥100 pg/mL) (Table 2, Figure 1). Mean androstenedione and gonadotropins levels were in the normal range. Nevertheless, in the TdP group was found an increased prevalence of subjects with suppressed LH, as well as a significantly lower FSH concentration when compared to control subjects (Table 2).

Whereas hypogonadism was a very common finding (23 of 26, ~90% of cases), underlying pathogenesis was very heterogeneous (Supplemental Table 2). In fact, as indicated by the associated gonadotropins levels, hypogonadism was primary/peripheral in 6 patients (26%, high gonadotropins), secondary/central in 5 (22%, low gonadotropins), and mixed in 12 (52%, gonadotropins normal in absolute, but inappropriately low for circulating testosterone). Among peripheral forms, 1 was due to bicalutamide treatment for prostate cancer, whereas in the other 5 patients, no evident causes were present (likely testicular senile involution or subclinical diseases). Central hypogonadism was drug-induced in 3 cases (gonadotrophin-releasing hormone-receptor agonists/antagonists for prostate cancer), but apparently unexplained in 2 patients (likely subclinical hypothalamic/pituitary diseases). Finally, mixed forms were due to active systemic inflammation^21,22^ in 9 patients and to chronic opioid therapy^23^ in 3 (Supplemental Table 2). A similar distribution of forms and causes was found also when TdP patients with severe hypogonadism were selectively considered (Supplemental Table 2).

It is important to note that, whereas testosterone was consistently lower than in control subjects throughout the 3 groups, only patients with mixed hypogonadisms showed circulating 17-β estradiol levels significantly increased when compared to control subjects (Supplemental Figure 3). This finding indicates that an increased peripheral androgen-to-estrogen conversion is a specific feature of TdP patients presenting with this type of hypogonadism, contributing to explaining testosterone lowering.

### SEX HORMONES LEVELS IN FEMALE SUBJECTS.

**FIGURE 2:**
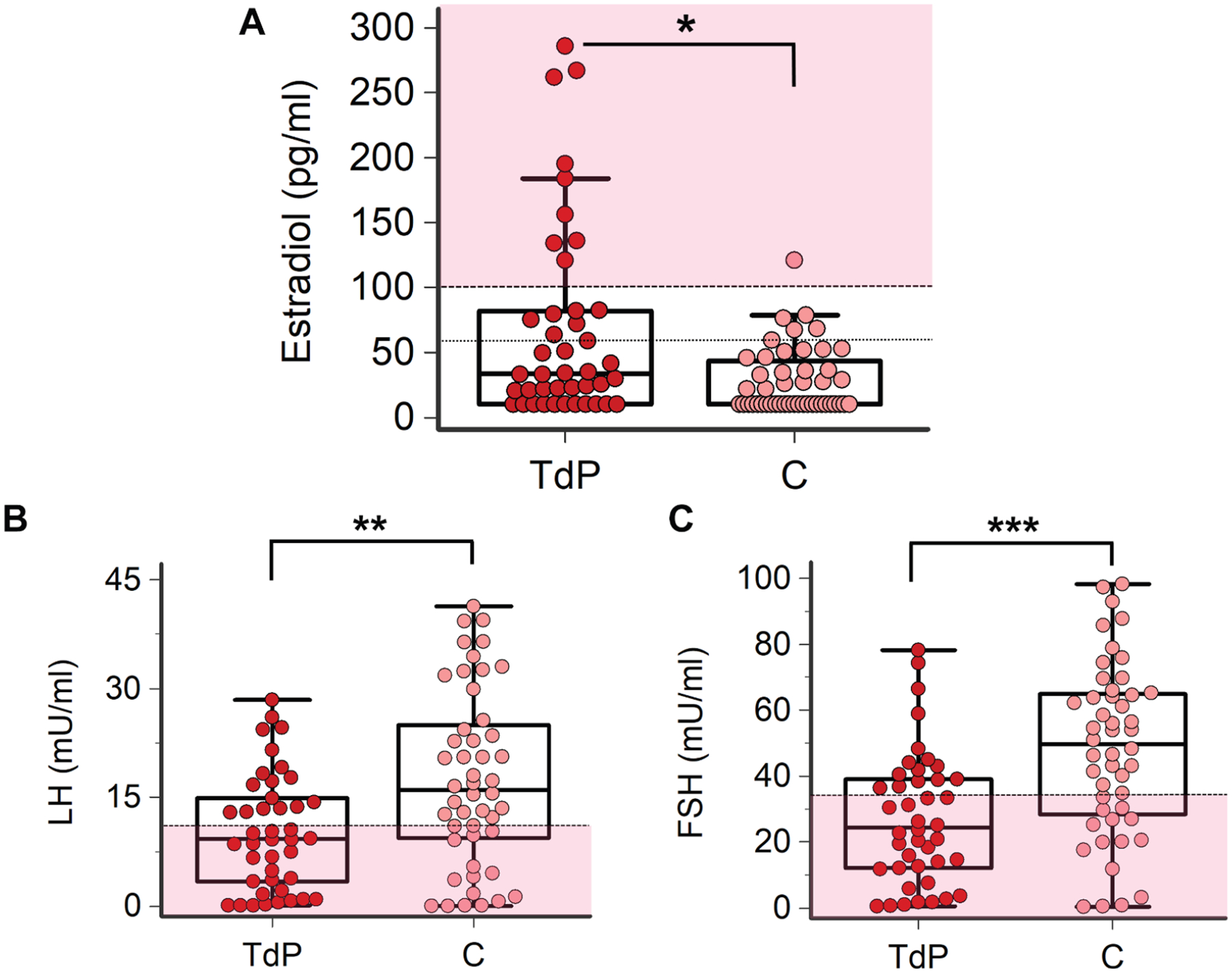
Circulating 17-β Estradiol and Gonadotropins Levels in Female Patients With TdP and Controls Subjects TdP patients, n = 42; C, n = 49. (A) 17-β estradiol: horizontal black dotted line indicates the upper limit of reference values in postmenopausal women (ie, 60 pg/mL); horizontal dotted line with pink above indicates values included in a premenopausal female range (ie, ≥100 pg/mL). (B) Luteinizing hormone (LH): horizontal dotted line with pink below indicates the lower limit of reference values in postmenopausal women (ie, 11 mU/mL), corresponding to the upper limit of reference values in premenopausal women. (C) Follicle stimulating hormone (FSH): horizontal dotted line with pink below indicates lower limit of reference values in postmenopausal women (ie, 35 mU/mL), corresponding to the upper limit of reference values in premenopausal women. Two-tailed Mann-Whitney *U* test or 2- tailed unpaired Student’s *t*-test: *P < 0.05, **P < 0.01, ***P < 0.001. Abbreviations as in Figure 1.

When compared to comorbidity control subjects, female TdP patients presented with significantly higher 17-β estradiol levels (~2×, with mean levels over the upper reference limit), and increased prevalence of hyperestrogenism (Table 3, Figure 2). Specifically, marked hyperestrogenism (ie, ≥100 pg/mL as usually only observed in premenopausal women) was 10× more common in TdP patients than in control subjects (21% vs 2%; *P* < 0.01). At the same time, TdP women showed significantly lower gonadotropins levels than control subjects did, with mean levels below reference values for postmenopausal female subjects. Accordingly, low LH and/or FSH were found in ~60% of TdP patients (Table 3). Although mean levels of androstenedione, testosterone (total and free), and progesterone levels were in both groups comprised within reference values, all these hormones were comparatively higher in TdP vs comorbidity control subjects (Table 3). Moreover, 17-β estradiol, androstenedione, and testosterone inversely correlated with gonadotropins levels (both FSH [17-β estradiol: *r* = −0.32, *P* < 0.05; androstenedione: *r* = −0.37, *P* < 0.05; testosterone: *r* = −0.44, *P* < 0.01] and LH [androstenedione: *r* = −0.36, *P* < 0.05; testosterone: *r* = −0.33, *P* < 0.05]; Spearman rank test).

### SEX HORMONES LEVELS AND ARRHYTHMIA OUTCOME IN TdP PATIENTS.

Over one-half of the TdP patients (36 of 68, 53%) experienced an adverse arrhythmia outcome: they developed VF/SCA and/or underwent electric shock (complicated TdP).

#### Male subjects.

TdP was complicated in 14 of 26 men (54%; TdP rapidly degenerated to VF/SCA, n = 6; in-/out-of-hospital VF/SCA followed by direct current shock, only later revealed to be a manifestation of TdP episodes, n = 4; direct current shock for sustained TdP not responsive to medical therapy, n = 4).

**FIGURE 3:**
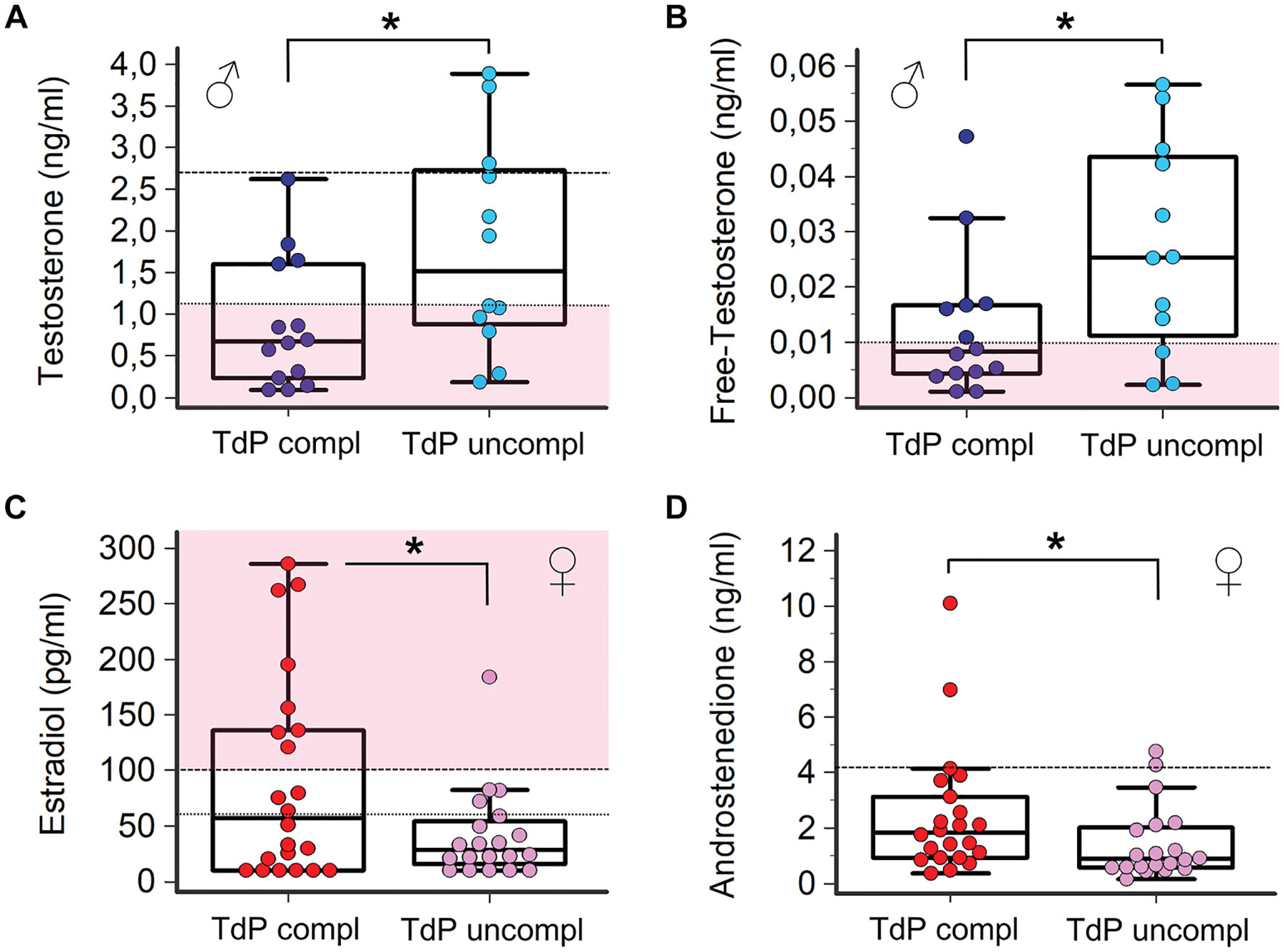
Circulating Sex Hormones Levels and Arrhythmia Outcome in TdP Patients (A,B) Total and free testosterone in male patients with complicated (compl) (n = 14) vs uncomplicated (uncompl) (n = 12) TdP. Horizontal black dotted line indicates the lower limit of reference values in men (total testosterone: 2.7 ng/mL; free testosterone: 0.065 ng/mL); horizontal dotted line with pink below indicates the upper limit of reference values in premenopausal women (total testosterone: 1.1 ng/mL; free testosterone: 0.01 ng/mL). (C,D) 17-β estradiol and androstenedione in female patients with complicated (n = 22) vs uncomplicated (n = 20) TdP. 17-β estradiol: horizontal black dotted line indicates the upper limit of reference values in postmenopausal women (ie, 60 pg/mL); horizontal dotted line with pink above indicates values included in a premenopausal female range (ie, ≥100 pg/mL). Androstenedione: horizontal black dotted line indicates the upper limit of reference values in women (ie, 4.1 ng/mL). Two-tailed Mann-Whitney *U* test: *P < 0.05. Abbreviations as in Figure 1.

Although comparable for age, patients with complicated TdP showed significantly lower testosterone levels, approximately 50% reduction, with respect to those with uncomplicated TdP (total: 0.87 ± 0.77 vs 1.80 ± 1.27 ng/mL, *P* < 0.05; free: 0.013 ± 0.013 vs 0.027 ± 0.019 ng/mL, *P* < 0.05) (Supplemental Table 3, Figures 3A and 3B). On the contrary, no significant differences between the 2 groups were observed in terms of age, gonadotropins and 17-β estradiol, or other sex hormones (Supplemental Table 3).

#### Female subjects.

An adverse TdP outcome was observed in 22 of 42 women (52%; TdP rapidly degenerated to VF/SCA, n = 9; in-/out-of-hospital VF/SCA followed by direct current shock, only later revealing to be a manifestation of TdP episodes, n = 5; direct current shock for sustained TdP not responsive to medical therapy, n = 8).

When compared to uncomplicated patients, subjects with complicated TdP presented with higher 17-β estradiol levels (>2×; 90.6 ± 91.7 vs 41.6 ± 41.0 ng/mL, *P* < 0.05), along with an increased prevalence of marked hyperestrogenism (36% vs 5%, *P* < 0.05): 8 of 9 cases with 17-β estradiol $100 pg/mL observed in the whole TdP cohort belonged to the complicated group (Supplemental Table 4, Figure 3C). Patients with complicated TdP also showed increased levels of androstenedione (2.45 ± 2.30 vs 1.42 ± 1.32 ng/mL, *P* < 0.05) (Supplemental Table 4, Figure 3D), whereas no differences were found between the 2 groups in terms of age, gonadotropins, or other sex hormones (Supplemental Table 4). Notably, in complicated TdP women, androstenedione levels robustly correlated with those of 17-β estradiol (*r* = 0.52, *P* < 0.05), as well as of testosterone (*r* = 0.73, *P* < 0.001) (Supplemental Figure 4). Moreover, in this subgroup a significant inverse correlation among 17-β estradiol, androstenedione, and testosterone with gonadotropins levels was also observed (FSH [17-β estradiol: *r* = −0.41, *P* < 0.05; androstenedione: *r* = −0.51, *P* < 0.05; testosterone: *r* = −0.52, *P* < 0.05] and LH [androstenedione: *r* = −0.45, *P* = 0.038; testosterone: *r* = −0.54, *P* = 0.010]; Spearman rank test) (Supplemental Figures 5 and 6).

### IN VITRO IMPACT OF SEX HORMONES PROFILES OBSERVED IN MALE AND FEMALE TdP PATIENTS ON GUINEA PIG VENTRICULAR MYOCYTE APD.

**FIGURE 4:**
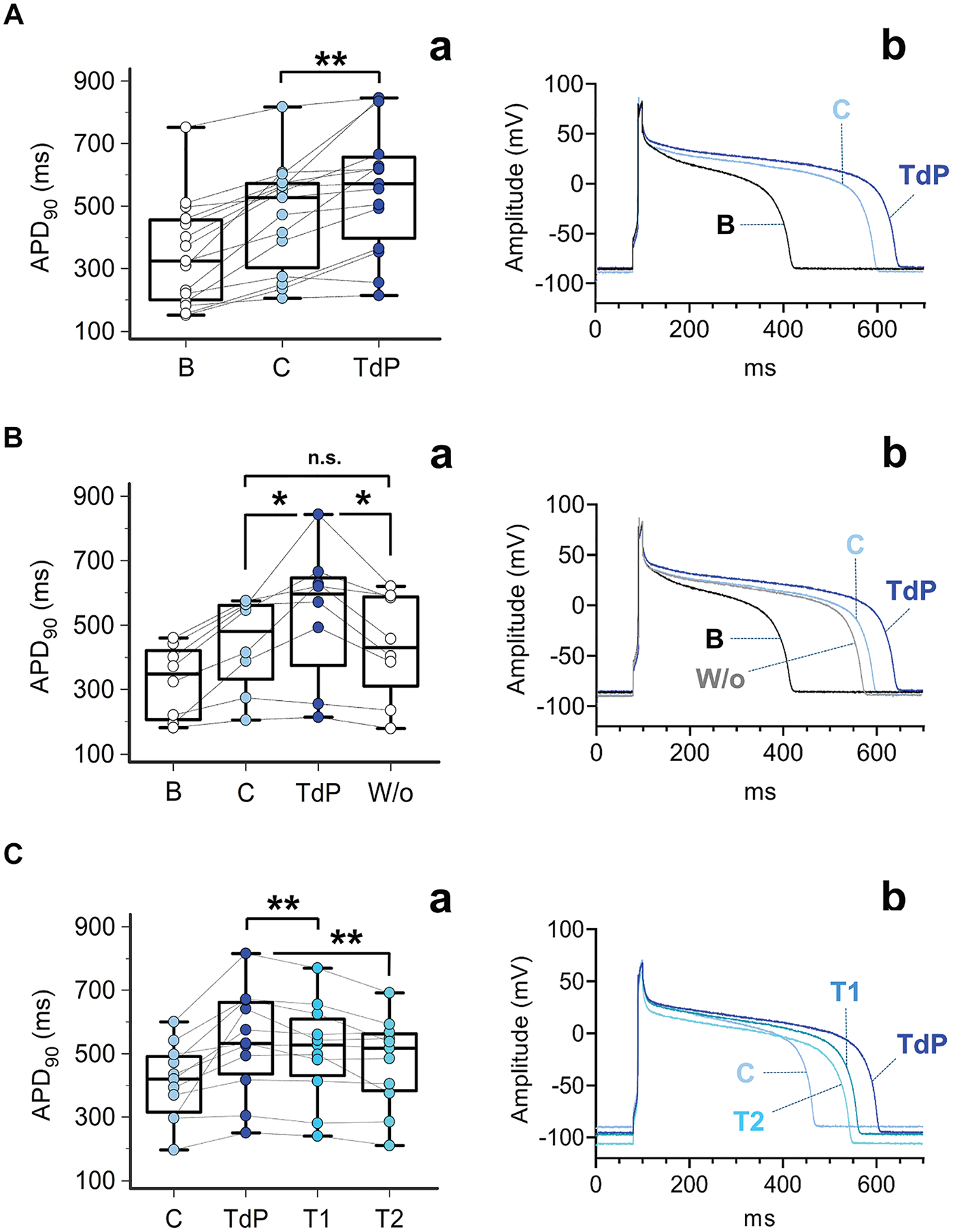
In Vitro Impact of Sex Hormones Profiles Observed in Male Patients With TdP and Control Subjects on Male Guinea Pig Ventricular Myocyte APD and Modulatory Activity of Increasing Testosterone Concentrations (A) (a) Action potential duration at 90% (APD_90_) measured in male guinea pig ventricular myocytes (guinea pigs, n = 3; ventricular myocytes, n = 15) perfused with regular Tyrode solution (baseline [B]), the sex hormones profile observed in male control subjects (C) (testosterone 4 ng/mL + 17-β estradiol 10 pg/mL + progesterone 0.2 ng/mL), and the sex hormones profile observed in male TdP patients (TdP) (testosterone 0.1 ng/mL + 17-β estradiol 100 pg/mL + progesterone 0.2 ng/mL). (b) Representative traces of male guinea pig ventricular myocyte APD_90_ after different treatments. (B) (a) APD_90_ measured in male guinea pig ventricular myocytes (guinea pigs, n = 2; ventricular myocytes, n = 8) perfused with regular Tyrode solution (baseline), the sex hormones profile observed in male control subjects (testosterone 4 ng/mL + 17-β estradiol 10 pg/mL + progesterone 0.2 ng/mL), the sex hormones profile observed in male TdP patients (testosterone 0.1 ng/mL + 17-β estradiol 100 pg/mL + progesterone 0.2 ng/mL), and after wash-out (W/o). (b) Representative traces of male guinea pig ventricular myocyte APD_90_ after different treatments along with the wash-out trace. (C) (a) APD_90_ measured in male guinea pig ventricular myocytes (guinea pigs, n = 2; ventricular myocytes, n = 12) perfused with the sex hormones profile observed in male control subjects (testosterone 4 ng/mL + 17-β estradiol 10 pg/mL + progesterone 0.2 ng/mL), the sex hormones profile observed in male TdP patients (testosterone 0.1 ng/mL + 17-β estradiol 100 pg/mL + progesterone 0.2 ng/mL), and the sex hormones profile observed in male TdP patients but with increasing testosterone concentrations (Treatment 1 [T1]: testosterone 4 ng/mL + 17-β estradiol 100 pg/mL + progesterone 0.2 ng/mL; Treatment 2 [T2]: testosterone 8 ng/mL + 17-β estradiol 100 pg/mL + progesterone 0.2 ng/mL). (b) Representative traces of male guinea pig ventricular myocyte APD_90_ after different treatments. Repeated measures analysis of variance, in all cases *P* < 0.001; post hoc multiple paired Student’s *t*-test with false discovery rate correction: *P < 0.05, **P < 0.01. NS = not significant; other abbreviations as in Figure 1.

**FIGURE 5:**
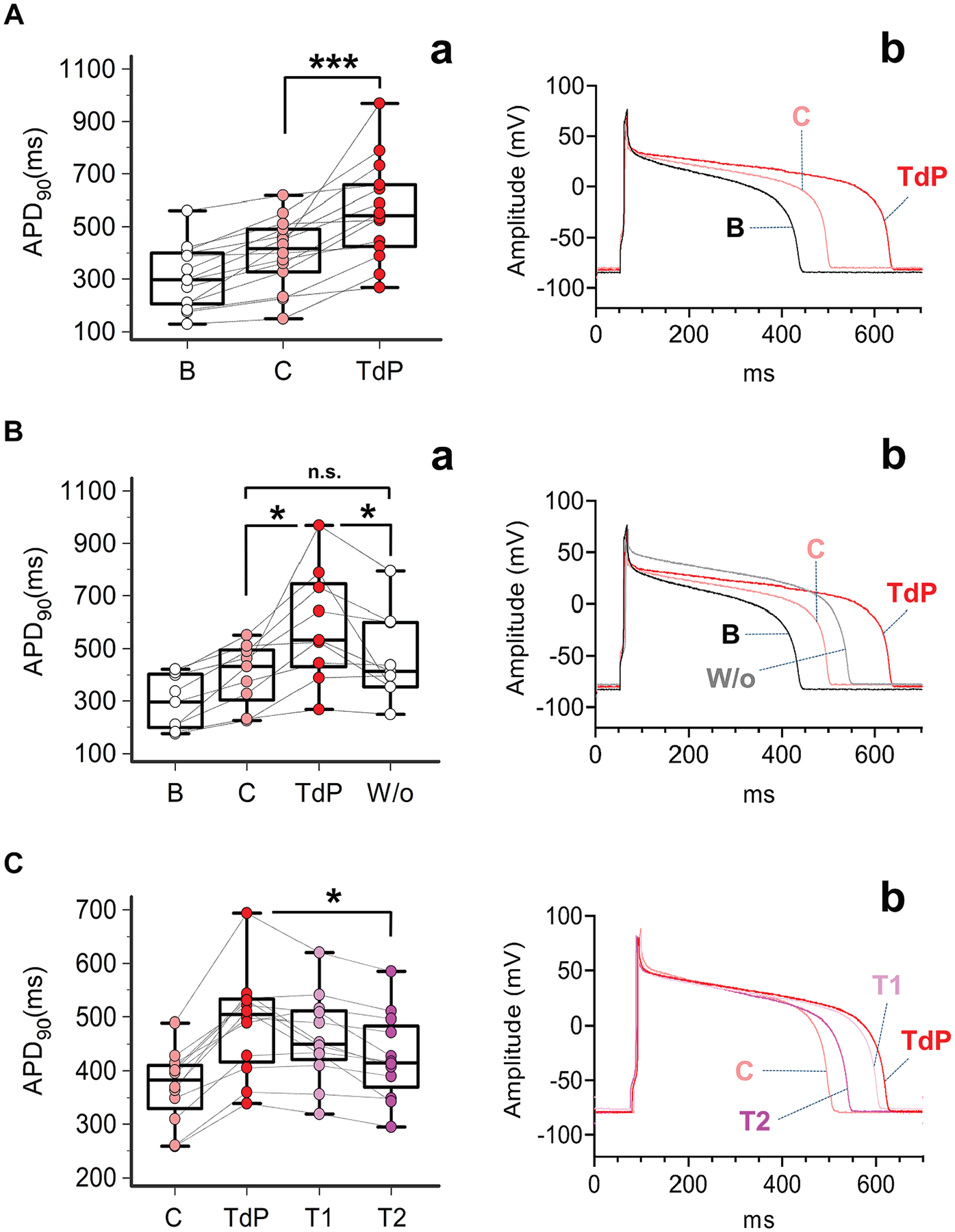
In Vitro Impact of Sex Hormones Profiles Observed in Female Patients With TdP and Control Subjects on Female Guinea Pig Ventricular Myocyte APD, and Modulatory Activity of Increasing Progesterone Concentrations (A) (a) APD_90_ measured in female guinea pig ventricular myocytes (guinea pigs, n = 3; ventricular myocytes, n = 14) perfused with regular Tyrode solution (baseline [B]), the sex hormones profile observed in female control subjects (C) (testosterone 0.1 ng/mL + 17-β estradiol 10 pg/mL + progesterone 0.2 ng/mL), and the sex hormones profile observed in female TdP patients (TdP) (testosterone 0.5 ng/mL + 17-β estradiol 150 pg/mL + progesterone 0.3 ng/mL). (b) Representative traces of female guinea pig ventricular myocyte APD_90_ after different treatments. (B) (a) APD_90_ measured in female guinea pig ventricular myocytes (guinea pigs, n = 2; ventricular myocytes, n = 9) perfused with regular Tyrode solution (baseline), the sex hormones profile observed in female control subjects (testosterone 0.1 ng/mL + 17-β estradiol 10 pg/mL + progesterone 0.2 ng/mL), the sex hormones profile observed in female TdP patients (testosterone 0.5 ng/mL + 17-β estradiol 150 pg/mL + progesterone 0.3 ng/mL), and after wash-out. b. Representative traces of female guinea pig ventricular myocyte APD_90_ after different treatments along with the wash-out trace. (C) (a) APD_90_ measured in female guinea pig ventricular myocytes (guinea pigs, n = 2; ventricular myocytes, n = 13) perfused with the sex hormones profile observed in female control subjects (testosterone 0.1 ng/mL + 17-β estradiol 10 pg/mL + progesterone 0.2 ng/mL), the sex hormones profile observed in female TdP patients (testosterone 0.5 ng/mL + 17-β estradiol 150 pg/mL + progesterone 0.3 ng/mL), and the sex hormones profile observed in male TdP patients but with increasing progesterone concentrations (Treatment 1 [T1]: testosterone 0.5 ng/mL + 17-β estradiol 150 pg/mL + progesterone 5 ng/mL; Treatment 2 [T2]: testosterone 0.5 ng/mL + 17-β estradiol 150 pg/mL + progesterone 20 ng/mL). (b) Representative traces of female guinea pig ventricular myocyte APD_90_ after different treatments. Repeated measures analysis of variance, in all cases *P* < 0.001; post hoc multiple paired Student’s *t*-test with false discovery rate correction: *P < 0.05, ***P < 0.001. Abbreviations as in Figures 1 and 4.

To evaluate whether sex hormones profiles observed in vivo in male and female patients with TdP significantly affect cardiac electrophysiology by increasing the propensity to TdP development, the acute effect of a combination of clinically comparable concentrations of testosterone, 17-β estradiol, and progesterone on APD was investigated in sex-matched guinea pig ventricular myocytes (Figures 4 and 5). Specifically, in order to reproduce hormonal levels really operating in the clinical setting and at the same time emphasize differences between patients and control subjects, the highest (75th, Q3) or the lowest (25th, Q1) quartile of concentration measured in vivo was selected for each hormone that was found to be increased or reduced in male or female complicated TdP patients vs control subjects, respectively. In the case of progesterone, which did not show any significant difference between patients and control subjects, a concentration as similar as possible to mean levels observed in vivo was used in male subjects (Supplemental Table 5).

#### Male subjects.

After recording APD_90_ under basal condition, male guinea pig myocytes were added with the sex hormones profile observed in male control subjects (testosterone 4 ng/mL + 17-β estradiol 10 pg/mL + progesterone 0.2 ng/mL) and then with the sex hormones profile observed in male TdP patients (testosterone 0.1 ng/mL + 17-β estradiol 100 pg/mL + progesterone 0.2 ng/mL). By comparing these 2 treatments, a significant mean APD_90_ increase was observed in TdP vs control conditions (+88.5 ms, from 469.4 ± 172.4 ms to 557.9 ± 200.2 ms, *P* = 0.007; n = 15) (Figure 4A). In a subset of these cells, APD_90_ was also recorded after washing out the solution reproducing the male TdP patients’ sex hormones profile. As a result, a significant shortening of APD_90_ was observed (−104.4 ms, from 536.5 ± 211.1 ms to 432.1 ± 164.7 ms, *P* = 0.012; n = 8), until values comparable with those measured in the presence of the male control subjects’ sex hormones profile (Figure 4B).

#### Female subjects.

Similarly, APD_90_ was recorded in female guinea pig myocytes, first under basal condition, after perfusion with the sex hormones profile observed in female control subjects (testosterone 0.1 ng/mL + 17-β estradiol 10 pg/mL + progesterone 0.2 ng/mL) and then with the sex hormones profile observed in female TdP patients (testosterone 0.5 ng/mL + 17-β estradiol 150 pg/mL + progesterone 0.3 ng/mL). Exposure to the female TdP sex hormones profile was associated with a significant mean APD_90_ increase when compared to control treatment (+160.2 ms, from 398.8 ± 132.4 ms to 559.0 ± 190.6 ms, *P* < 0.001; n = 14) (Figure 5A), an effect that was significantly reversed by 50.8% on washing out the TdP solution (−120.9 ms, from 587.6 ± 217.3 ms to 466.7 ± 167.7 ms, *P* = 0.035; n = 9) (Figure 5B).

### IN VITRO IMPACT OF SEX HORMONES PROFILES OBSERVED IN MALE AND FEMALE TdP PATIENTS ON hiPSC-CMs’ APD.

To validate on a human model the data obtained in guinea pigs, we repeated the patch-clamp experiments using hiPSC-CMs derived from male and female subjects.

#### Male subjects.

After recording APs, APD_90_ measurements were performed in hiPSC-CMs under basal condition with the sex hormones profile observed in male control subjects (testosterone 4 ng/mL + 17-β estradiol 10 pg/mL + progesterone 0.2 ng/mL) and then with the sex hormones profile observed in male TdP patients (testosterone 0.1 ng/mL + 17-β estradiol 100 pg/mL + progesterone 0.2 ng/mL). Mean APD_90_ showed a significant increase when control and TdP conditions were compared (+72.4 ms, from 476.6 ± 103.5 ms to 549.0 ± 142.8 ms, *P* = 0.022; n = 5) (Supplemental Figures 7A and 7B), substantially overlapping that observed in male guinea pig myocytes.

#### Female subjects.

APD_90_ was measured in hiPSC-CMs, first under basal condition, after perfusion with the sex hormones profile observed in female control subjects (testosterone 0.1 ng/mL + 17-β estradiol 10 pg/mL + progesterone 0.2 ng/mL) and then with the sex hormones profile observed in female TdP patients (testosterone 0.5 ng/mL + 17-β estradiol 150 pg/mL + progesterone 0.3 ng/mL). Female TdP sex hormones profile was associated with a significant mean APD_90_ increase when compared to control profile (+178.0 ms, from 383.4 ± 40.5 ms to 561.4 ± 72.8 ms, *P* < 0.001; n = 5) (Supplemental Figures 8A and 8B), an effect again very similar to that measured in female guinea pig myocytes.

Based on this evidence indicating a substantial overlap between the 2 experimental models, further experiments were performed on guinea pig cardiomyocytes only.

### IN VITRO EFFECT OF SEX-SPECIFIC HORMONAL INTERVENTIONS IN REVERTING TdP PROFILE- ASSOCIATED GUINEA PIG VENTRICULAR MYOCYTE APD PROLONGATION.

Given that the previous data support the hypothesis that both male and female TdP sex hormones profiles observed in vivo significantly prolong APD in guinea pig ventricular myocytes, further in vitro experiments were performed to evaluate whether sex-specific hormonal interventions may reverse these changes, specifically by increasing concentrations of testosterone in male subjects and progesterone in female subjects, respectively. In fact, because most male TdP patients were hypogonadal, increasing testosterone concentrations in vitro until reproducing normal blood values observed in vivo is the more reasonable and clinically practicable intervention. Whereas hyperestrogenism was the most common abnormality found in TdP female patients, anti-estrogenic therapies in these patients are not feasible for the associated significant risk of adverse effects.^24^ Conversely, progesterone is an attractive therapeutic option because: 1) it is commonly used also in young women owing to the favorable safety profile;^25,26^ and 2) its electrophysiological properties could counterbalance the APD-prolonging effects of 17-β estradiol excess.^4^ Moreover, to further mimic conditions really operating in the clinical setting, sex hormone concentrations consistent with blood level ranges reached during standard treatments were used: 4 or 8 ng/mL for testosterone for male subjects (transdermal testosterone, 50 mg [3.3–3.9 ng/mL] or 100 mg [6.8–9.3 ng/mL] once daily),^9,10,27^ and 5 or 20 ng/mL for progesterone for female subjects (oral progesterone, 200 mg [2.8–4.7 ng/mL] or 400 mg [16.2–21.2 ng/mL] once daily),^8,9,28,29^ respectively.

#### Male subjects.

After recording APD_90_ in control (testosterone 4 ng/mL + 17-β estradiol 10 pg/mL + progesterone 0.2 ng/mL) and TdP (testosterone 0.1 ng/mL + 17-β estradiol 100 pg/mL + progesterone 0.2 ng/mL) conditions, male guinea pig myocytes were perfused with the same TdP solution, but with 2 increasing testosterone concentrations: 4 ng/mL (T1: testosterone 4 ng/mL + 17-β estradiol 100 pg/mL + progesterone 0.2 ng/mL) and 8 ng/ml (T2: testosterone 8 ng/mL + 17-β estradiol 100 pg/mL + progesterone 0.2 ng/mL). Both treatments resulted in a significant dose-dependent APD_90_ decrease when compared to TdP, reversing the effect by 24.1% (T1: −29.8 ms, from 557.2 ± 175.3 ms to 527.4 ± 161.6 ms, *P* = 0.009; n = 12) and 46.5% (T2: −57.5 ms, from 557.2 ± 175.3 ms to 499.7 ± 161.3 ms, *P* = 0.004; n = 12), respectively.

#### Female subjects.

Also in this case, APD_90_ was first recorded in control (testosterone 0.1 ng/mL + 17-β estradiol 10 pg/mL + progesterone 0.2 ng/mL) and TdP (testosterone 0.5 ng/mL + 17-β estradiol 150 pg/mL + progesterone 0.3 ng/mL) conditions. Then, female guinea pig myocytes were perfused with the same TdP solution, but with 2 increasing progesterone concentrations: 5 ng/mL (T1: testosterone 0.5 ng/mL + 17-β estradiol 150 pg/mL + progesterone 5 ng/mL) and 20 ng/mL (T2: testosterone 0.5 ng/mL + 17-β estradiol 150 pg/mL + progesterone 20 ng/mL). Treatment with the higher progesterone concentration (20 ng/mL) was associated with a significant APD_90_ shortening when compared to TdP, reversing the effect by 43.9% (T2: −51.9 ms, from 496.8.2 ± 97.0 ms to 444.9 ± 104.6 ms, *P* = 0.021; n = 13). Conversely, although a trend was observed, the lower progesterone concentration (5 ng/mL) did not significantly reverse the TdP effect (T1: −21.2 ms, from 496.8.2 ± 97.0 ms to 475.6 ± 95.3 ms, *P* = 0.11; n = 13).

## DISCUSSION

The main findings of the present study are the following. 1) When compared to control subjects, a differential sex hormone profile was observed in TdP men and women, primarily low testosterone in male subjects, and high 17-β estradiol with reduced gonadotropins in female subjects. 2) Within the TdP cohort, lower testosterone in men and higher 17-β estradiol in women are associated with a worse outcome for the arrhythmia (ie, degeneration to VF/SCA and/or necessity of electric shock). 3) In vitro reproduction of sex-specific TdP hormone profiles prolonged APD in sex-matched guinea pig ventricular myocytes and hiPSC-CMs, an effect that is reversed by the addition of testosterone in male subjects and progesterone in female subjects, respectively (Central Illustration).

In recent years, an accumulating body of evidence points to important sex-related differences in arrhythmic risk, at least in part driven by the complex effects exerted by sex hormones on cardiac ion channels.^30,31^ In particular, it is well demonstrated that adult women are characterized by a greater risk of LQTS and related malignant arrhythmias, specifically TdP, when compared to men.^2,30,31^ Moreover, it is increasingly documented that hypogonadism in male subjects is associated with an enhanced susceptibility to LQTS/TdP.^21,32,33^ However, information on actual levels of sex hormones present in TdP patients at the moment of arrhythmia are limited,^11^ and this gap of knowledge could explain why these emerging concepts are to date poorly translated into the clinical practice. In fact, sex hormones are not routinely measured, nor do they represent a major therapeutic target in patients who develop TdP.

In the present study, we provide evidence for the first time that sex hormone imbalances are a common feature in TdP patients, both male and female, but with distinctive sex-related profiles. Specifically, men with TdP showed significantly lower testosterone levels, less than one-half of comorbidity control subjects. In approximately two-thirds of all cases, testosterone deficiency was severe, with levels as low as those usually observed in premenopausal female subjects. The relevance of these abnormalities is strengthened by the evidence that biologically active free testosterone (ie, SHBG unbound) was in parallel reduced. Whereas these data provide evidence that a large percentage of TdP male subjects were profoundly hypogonadic when the arrhythmia occurred, underlying causes of hypogonadism in our cohort were very different and included central forms owning to depressed production of gonadotropins, peripheral forms due to primary testicular causes, or mixed forms in which both the mechanisms were concomitantly involved. Active inflammatory processes and medications (androgen-deprivation therapy, opioids) were the most frequent causative factors, although in approximately one-third of cases etiology remained unknown. Overall, this scenario suggests that independent of the specific mechanism involved, the “feminization” of the patient is the key pathogenic factor that, by removing the QT-shortening effect of testosterone, can enhance the predisposition of men to LQTS/TdP to a similar extent to what is usually observed in women. The important impact of these changes in the clinical setting is further and remarkably supported by the evidence here reported that testosterone levels were particularly reduced in those patients who experienced a short-term adverse arrhythmia outcome–degeneration to VF and cardiac arrest and/or necessity of DC shock. We found that all these complicated men were invariably hypogonadic, and in most cases severely hypogonadic, with mean testosterone levels falling in the range of premenopausal female subjects.

Furthermore, we observed that in one-half of TdP male subjects, hypotestosteronemia occurred in combination with increased 17-β estradiol levels and not infrequently (~25%) very high concentrations as are normally found in premenopausal female subjects only. This additional hormonal imbalance was specifically found in TdP patients presenting with mixed hypogonadism, in turn being in most cases the result of active inflammatory states or methadone maintenance therapy. It has been demonstrated that both cytokines and opioids not only can inhibit gonadotropin secretion via direct effects on anterior pituitary and/or hypothalamus, but also enhance peripheral androgen-to-estrogen conversion by increasing the expression of aromatase which catalyzes the biotransformation of testosterone to estradiol.^21–23,34,35^ Given that estradiol can prolong APD in vitro and QTc in vivo,^4,31^ it is conceivable that in this subgroup of patients the concomitant hyperestrogenic state may have contributed to TdP development by synergistically operating with hypotestosteronemia.

The most striking feature observed in women who developed TdP is the presence of significantly increased 17-β estradiol levels, doubled when compared to comorbidity control subjects. Moreover, the prevalence of hyperestrogenism in TdP female subjects was almost 4× higher than in control subjects, a difference rising up to 10× when values ≥100 pg/mL (marked hyperestrogenism) were specifically considered. Remarkably, the highest 17-β estradiol concentrations were observed in patients who experienced an adverse arrhythmia outcome, a finding strongly supporting the clinical relevance of this alteration. At the same time, TdP women showed reduced levels of gonadotropins, with mean concentrations falling in a range usually observed in premenopausal female subjects (where physiologically high 17-β estradiol levels inhibit the hypothalamus/anterior pituitary axis).^36^ Although these alterations are in some way unexpected given the advanced age of these patients (on average >80 years), on the other hand they fit well with the notion that estrogens can promote APD/QTc prolongation, thereby with the hypothesis that women who unusually preserve a significant 17-β estradiol production over time (“super-females”) may be more susceptible to TdP development. Putative underlying mechanisms might involve an abnormally prolonged preservation of the ovarian function and/or an enhanced production in extragonadal tissues such as the adrenal glands. The evidence here provided that TdP women, particularly those with complicated forms, show androstenedione levels higher than control subjects seems to suggest a prevalent role for the second mechanism. Androstenedione, which is predominantly secreted by the adrenal cortex, is a crucial sex-steroid precursor primarily involved in the synthesis of testosterone (directly) and estradiol (indirectly, via testosterone or estrone formation), but also progesterone.^37^ In agreement, all these hormones were higher in TdP female subjects than in control subjects, with a direct correlation existing between the levels of androstenedione and those of testosterone and estradiol, respectively. At the same time, androstenedione, testosterone, and estradiol concentrations were inversely correlated with circulating gonadotropins, thereby suggesting a role for the elevation of these hormones in inhibiting the hypothalamus/anterior pituitary axis.

The other key information deriving from this study is that the sex-specific hormone profiles demonstrated in vivo in our patients significantly affect ventricular myocyte electrophysiology in vitro leading to an increased TdP susceptibility, and that these changes can be effectively reversed by sex-specific hormone-modulatory interventions. We provided evidence that acute exposure of sex-matched guinea pig ventricular myocytes or hiPSC-CMs to sex hormones profiles (testosterone + 17-β estradiol + progesterone) at concentrations strictly mimicking those really observed in male and female patients with TdP was associated with a significant prolongation of the APD when compared to control sex hormones profiles, and that such effects were rapidly and significantly reversed on washing out the TdP solution, or by increasing in the TdP solution the concentrations of testosterone in male subjects and progesterone in female subjects, respectively. These findings have 2 main clinical implications. First, they provide strong support to the view that the sex hormones imbalances found in our patients can, already in the short term, significantly contribute to prolong QTc in vivo, in turn representing the key electrophysiological abnormality promoting TdP occurrence. This effect is expected to be additive with any other concomitant QT-prolongation risk factors present in a given patient, thereby contributing to increase the risk of developing critical QTc prolongation precipitating the onset of TdP (multihit theory).^20,38,39^ A nontranscriptional regulation of cardiac repolarization currents by sex hormones is the most likely underlying mechanism accounting for the acute changes observed. Accordingly, it has been demonstrated that either testosterone and progesterone rapidly (minutes) shortened APD in isolated guinea pig ventricular myocytes by enhancing I_Ks_ and suppressing I_CaL_, probably either through a receptor-independent membrane-localized androgen receptors.^40,41^ Moreover, by using Langendorff-perfused guinea pig hearts, patch-clamped guinea pig cardiomyocytes and culture cells over-expressing the human ether-a-go-go-related gene channel, Kurokawa et al.^42^ provided evidence that 17-β estradiol acutely prolongs APD and QTc as a result of a direct interference in the channel gating leading to I_Kr_ inhibition. Although significant, these changes probably underestimate the overall impact of sex hormones on cardiac electrophysiology in vivo. Besides such acute nontrascriptional effects, there is evidence the testosterone, 17-β estradiol and progesterone can also chronically modulate cardiac repolarization currents via genomic pathways, involving nuclear translocation and regulation of gene expression,^43,44^ possibly enhancing in the medium-long term the clinical significance of these changes.

Second, the evidence here provided that increasing the concentrations of testosterone in male subjects or progesterone in female subjects was effective in reversing APD prolongation induced in vitro by the TdP solution, points to these sex-specific hormone interventions as attractive novel treatments for a number of patients with LQTS/TdP. In particular, the fact that the hormonal concentrations demonstrated to be effective in vitro were those really observed in the blood of humans under standard (and well-tolerated) treatments with testosterone or progesterone, not only suggests the feasibility of this approach in the clinical practice, but also provide specific target levels (testosterone 4–8 ng/mL in male subjects; progesterone 5–20 ng/mL in female subjects) that may be monitored to obtain the best effectiveness/safety ratio in the single patient. Except for specific absolute contraindications, fundamentally a history of prostatic cancer^44^ for testosterone-replacement therapy, large randomized trials and meta-analyses provided evidence for the long-term oncologic and cardiovascular safety of these treatments,^45–47^ particularly when circulating hormonal levels are maintained in a comparable range to our study.^45^

Finally, although progesterone treatment to counterbalance estradiol excess may not be clinically appropriate for men, it would be interesting to explore in the future whether it could exert an additive effect in reducing APD, alongside the restoration of testosterone to normal levels.

## CONCLUSIONS

The present study provides evidence that different sex hormone profiles, primarily low testosterone in male subjects and high 17-β estradiol in female subjects, are associated with TdP occurrence and outcome in men and women. These alterations, detected in a substantial proportion of TdP patients and reminiscent of conditions usually observed in premenopausal women only, can promote arrhythmogenesis at least in part via direct and reversible effects on cardiac electrophysiology. Overall, our findings support the view that an absolute (men) or relative (postmenopausal women) feminine shift in the sex hormone profile is an epidemiologically relevant risk factor for TdP development in both male subjects and female subjects. These data, which are in total agreement with the results of the Oregon SUDS study,^12^ also strongly suggest that the association found in such study between abnormal sex hormone levels and SCD in the general population may be, at least in part, due an increased incidence of LQTS/TdP. However, whether observed sex hormone profiles are causative of initial TdP episodes, or a subsequent risk factor for short-term recurrence, or both, it is not currently clear and require further investigation.

In a clinical perspective, the present work substantiates the recommendation to measure sex hormones levels in all patients who develop TdP, particularly when complicated or refractory to conventional treatments. It is likely that in several cases significant sex hormones imbalances are present, particularly hypogonadism in men or hyperestrogenism in women, that are susceptible to specific modulation by hormonal therapies. The administration of testosterone in male subjects and progesterone in female subjects could represent in these cases an important additional antiarrhythmic intervention potentially able to improve the short-term TdP outcome, as well as reduce the long-term risk of TdP recurrence. Whereas data from small clinical studies provide preliminary support to this hypothesis,^8–10^ large randomized controlled trials are warranted to confirm the efficacy and safety of hormonal treatment in these patients prior to further clinical application in this arena.

## Supplementary Material

1

## Figures and Tables

**TABLE 1:** Demographic, Clinical, and Laboratory Characteristics Of Patients With TdP Control Subjects

	Patients (n = 68)	Control Subjects (n = 77)	*P* Value
Age, y	81 (72.5–85)	78 (72–85)	0.61
Female	42/68 (62)	48/77 (62)	1.00
Male	26/68 (38)	29/77 (38)	1.00
Mean QTc, ms	592.9 ± 80.0	431.4 ± 31.0	<0.001
Mean QTc-prolonging risk factor number per patient^a^	4.9 ± 1.5		
Electrolyte imbalances	48/67 (71)		
Hypokalemia	34/48 (70)		
Hypocalcemia	23/48 (48)		
Hypomagnesemia	7/48 (15)		
Concomitant diseases^b^			
Cardiac diseases	56/68 (82)	59/77 (77)	0.42
Left ventricular hypertrophy	27/56 (48)	38/59 (65)	
Dilated cardiomyopathy/heart failure	23/56 (41)	17/59 (29)	
II-III degree atrioventricular block	21/56 (38)	0	
Acute coronary syndrome	11/56 (20)	0	
Chronic coronary artery disease	9/56 (16)	24/59 (41)	
Bradycardia < 50 beats/min	6/56 (11)	0	
Extra-cardiac diseases	27/68 (40)	28/77 (36)	0.73
Diabetes mellitus type II	21/27 (78)	15/28 (54)	
Chronic kidney disease	11/27 (41)	12/28 (43)	
Hypothyroidism	2/27 (7)	0	
Anorexia nervosa/starvation	2/27 (7)	0	
Anti-Ro/SSA positivity	21/38 (55)		
Systemic inflammation^c^	55/68 (81)		
C-reactive protein, mg/dL	2.10 (0.7–5.8)		
QTc-prolonging-medications^d^	42/68 (61)		
Amiodarone	17/42 (41)		
Citalopram	6/42 (14)		
Androgen deprivation therapy	4/42 (10)		
Clarithromycin	3/42 (7)		
Fluconazole	3/42 (7)		
Ciprofloxacin	3/42 (7)		
Azithromycin	2/42 (5)		
Levofloxacin	2/42 (5)		
Sotalol	2/42 (5)		
Haloperidol	2/42 (5)		
Methadone	2/42 (5)		
Mean medication number per patient	0.9 ±.0.8		

Values are mean ± SD, median (Q1-Q3), n, or n/N (%). Differences in continuous variables were evaluated by the 2-tailed unpaired Student’s *t*-test or the 2-tailed Mann-Whitney *U* test. Differences in categorical variables were evaluated by the 2-sided Fisher exact test.

a Including electrolyte imbalances, diseases, anti-Ro/SSA positivity, systemic inflammation, and QTc-prolonging medications.

b Diseases recognized to be a risk factor for QTc prolongation.

c Increased C-reactive protein level (>0.5 mg/dL) with or without a definite inflammatory disease.

d Medications with known or possible risk of TdP (as indicated by CredibleMeds). Serum potassium, calcium, or magnesium measurements available before replacement therapy in 63, 52, and 39 of 68 patients, respectively; anti-Ro/SSA antibodies tested in 38 of 68 patients.

SSA = Sjogren syndrome-related antigen A; TdP = torsades de pointes.

**TABLE 2:** Sex Hormones Levels in Male Patients With TdP Control Subjects

	TdP (n = 26)	Control Subjects (n = 29)	*P* Value
Total testosterone (rv, 2.7–10.9 ng/mL)	1.29 ± 1.12 ↓	2.91 ± 2.18	<0.001^a^
Subjects with low testosterone (≤2.7 ng/mL)	23/26 (88) ↑	16/29 (59)	0.017^a^
Subjects with very low testosterone (≤1.1 ng/mL)	16/26 (62) ↑	3/29 (10)	<0.001^a^
SHBG (rv, 10–57 nmol/L)	56.7 ± 28.8	45.2 ± 14.5	0.06
Free testosterone (rv, ≥0.065 ng/mL)	0.019 ± 0.014 ↓	0.047 ± 0.039	<0.001^a^
Subjects with very low free testosterone (≤0.01 ng/mL)	11/26 (42) ↑	0/29	<0.001^a^
Androstenedione (rv, 0.4–3.1 ng/mL)	1.54 ± 1.04	1.23 ± 0.75	0.65
17-β estradiol (rv, ≤50 pg/mL)	62.9 ± 56.0 ↑	28.1 ± 23.6	0.013^a^
Subjects with high 17-β estradiol (≥50 pg/mL)	13/26 (50) ↑	5/29 (17)	0.02^a^
Subjects with very high 17-β estradiol (≥100 pg/mL)	6/26 (23) ↑	0/29	0.0089^a^
Progesterone (rv, 0.3–0.9 ng/mL)	0.23 ± 0.23	0.16 ± 0.10	0.55
LH (rv, 0.8–8 mU/mL)	5.93 ± 6.79	6.79 ± 5.79	0.15
Subjects with low LH (≤0.8 mU/mL)	5/26 (19) ↑	0/29	0.019^a^
Subjects with high LH (≥8 mU/mL)	7/26 (27)	4/29 (14)	0.32
FSH (rv, 1.2–15.8 mU/mL)	8.51 ± 8.32 ↓	14.4 ± 12.2	0.01^a^
Subjects with low FSH (≤1.2 mU/mL)	1/26 (4)	0/29	0.47
Subjects with high FSH (≥15.8 mU/mL)	2/26 (8)	6/29 (21)	0.26

Values are mean ± SD or n/N (%). Differences in continuous variables were evaluated by the 2-tailed unpaired Student’s *t*-test, or the 2-tailed Mann-Whitney *U* test. Differences in categorical variables were evaluated by the 2-sided Fisher exact test. Arrows indicate statistically significant increase/decrease in TdP patients when compared to control subjects.

a *P* < 0.05.

FSH = follicle stimulating hormone; LH = luteinizing hormone; rv = reference values; other abbreviations as in Table 1.

**Table 3:** Sex Hormones Levels in Female Subjects With TdP Control Subjects

	TdP (n = 42)	Control Subjects (n = 49)	*P* Value
17-β estradiol (rv, <20–60 pg/mL)	67.3 ± 74.9 ↑	34.8 ± 52.4	0.013^a^
Subjects with high 17-β estradiol (>60 pg/mL)	15/42 (36) ↑	5/49 (10)	0.0048^a^
Subjects with very high 17-β estradiol (>100 pg/mL)	9/42 (21) ↑	1/49 (2)	0.0049^a^
Progesterone (rv, 0.1–1.1 ng/mL)	0.27 ± 0.34 ↑	0.15 ± 0.13	0.018^a^
Subjects with high progesterone (>1.1 ng/mL)	2/42 (4)	0/49 (0)	0.21
Total testosterone (rv, 0.1–1.1 ng/mL)	0.39 ± 0.45 ↑	0.23 ± 0.21	0.015^a^
Subjects with high testosterone (>1.1 ng/mL)	1/42 (2)	0/49 (0)	0.46
SHBG (rv, 18–144 nmol/L)	63.0 ± 42.2	69.2 ± 33.8	0.10
Free testosterone (rv, <0.01 ng/mL)	0.013 ± 0.045 ↑	0.003 ± 0.004	0.0094^a^
Subjects with high free testosterone (>0.01 ng/mL)	4/42 (10)	2/49 (4)	0.41
Subjects with very high free testosterone (>0.065 ng/mL)	0/42 (0)	0/49 (0)	1.00
Androstenedione (rv, 0.4–4.1 ng/mL)	1.96 ± 1.95 ↑	1.01 ± 1.06	0.001^a^
LH (rv, 11–40 mU/mL)	10.2 ± 8.0 ↓	17.2 ± 12.2	0.0021^a^
Subjects with low LH (<11 mU/mL)	25/42 (60) ↑	15/49 (31)	0.0066^a^
FSH (rv, 35–160 mU/mL)	27.2 ± 20.1 ↓	47.2 ± 26.4	<0.001^a^
Subjects with low FSH (<35 mU/mL)	27/42 (64) ↑	16/49 (33)	0.0033^a^

Values are mean ± SD or n/N (%). Differences in continuous variables were evaluated by the 2-tailed unpaired Student’s *t*-test, or the 2-tailed Mann-Whitney *U* test. Differences in categorical variables were evaluated by the 2-sided Fisher exact test. Arrows indicate statistically significant increase/decrease in TdP patients when compared to control subjects.

a *P* < 0.05.

FSH = follicle stimulating hormone; other abbreviations as in Tables 1 and 2.

## PERSPECTIVES

### COMPETENCY IN MEDICAL KNOWLEDGE:

An absolute (men) or relative (postmenopausal women) feminine shift in the sex hormone profile is an epidemiologically relevant risk factor for TdP development in both male subjects and female subjects. In a clinical perspective, the present work substantiates the recommendation to measure sex hormones levels in all patients who develop TdP, because administration of testosterone in male patients and progesterone in female patients could represent an important additional antiarrhythmic intervention.

### TRANSLATIONAL OUTLOOK:

Whereas data from small clinical studies provide preliminary support to this hypothesis, large randomized controlled trials are warranted to confirm the beneficial impact of hormonal treatment in these patients.

## APPENDIX

For supplemental methods, figures, tables, and references, please see the online version of this paper.

## ABBREVIATIONS AND ACRONYMS

APD: action potential duration
APD_90_: action potential duration at 90% full repolarization
FSH: follicle stimulating hormone
hiPSC-CM: human-induced pluripotent stem cell–derived cardiomyocyte
I_CaL_: L-type calcium current
I_Kr_: rapid component of the delayed-rectifier potassium current
I_Ks_: slow component of the delayed-rectifier potassium current
LH: luteinizing hormone
LQTS: long QT syndrome
SCA: sudden cardiac arrest
SCD: sudden cardiac death
T1/2: treatment 1/2
TdP: torsades de pointes
VF: ventricular fibrillation
