# Supplementary material for: Sex Hormone Profiles in Patients With Torsades de Pointes Ventricular Tachycardia: A Clinical-Electrophysiological Translational Study
Source: JACC Clin Electrophysiol. Author manuscript; Available in PMC 2026 May 5. (PMC13140104; doi:10.1016/j.jacep.2025.07.009)
Supplement: 1 [file NIHMS2169546-supplement-1.docx]

**SUPPLEMENTAL APPENDIX**

**-Supplemental Methods:** pages 2-6

**-Supplemental Table 1:** page 7

**-Supplemental Table 2:** page 8

**-Supplemental Table 3:** page 9

**-Supplemental Table 4:** page 10

**-Supplemental Table 5:** page 11

**-Supplemental Figure 1:** page 12

**-Supplemental Figure 2:** page 13

**-Supplemental Figure 3:** page 14

**-Supplemental Figure 4:** page 15

**-Supplemental Figure 5:** page 16

**-Supplemental Figure 6:** page 17

**-Supplemental Figure 7:** page 18

**-Supplemental Figure 8:** page 19

**-Legend to Supplemental Figures:** pages 20-22

**-References:** page 23

**SUPPLEMENTAL METHODS**

**Sex hormones measurement.** Testosterone (total), LH, and FSH were measured by UniCel DxI 800 (Beckman Coulter), while SHBG, androstenedione, progesterone, and estradiol levels by Immulite 2000 (Siemens). Free testosterone was calculated based on SHBG and total testosterone concentration. This parameter more accurately reflects the level of bioactive testosterone than does the sole measurement of total serum testosterone. Testosterone circulates in plasma unbound (free approximately 2-3%) and bound to SHBG. The SHBG-bound fraction is biologically inactive, because of the high binding affinity of SHBG for testosterone.

**ECG recordings.** The QT interval was manually measured on a standard 12-lead ECG, by the onset of the Q wave or the onset of the QRS complex to the end of the T wave, defined as the return to the T-P baseline. When prominent U waves (>1 mm) merging into T waves were present, they were included in the QT measurement.^1^ The QT interval, determined as the longest hand-measured QT interval in any lead, was corrected for heart rate by the Bazett’s formula [QT/RR interval^1/2^] to yield the QTc value. QTc was measured from 3 non-consecutive beats (mean value) by a single investigator (M.A.). QTc was considered prolonged if >470 ms in males or >480 ms in females, in accordance with the American Heart Association/American College of Cardiology (AHA/ACC) recommendations.^2^

**Isolation of ventricular myocytes from guinea-pigs.** Male and female Hartley guinea pigs were anesthetized with isoflurane in accordance with the IACUC approval of this study at the VA New York Harbor Healthcare System and conforming to the NIH guidelines. Hearts were rapidly excised and Langendorff perfused with Tyrode’s solution containing (in mM): 118 NaCl, 4.8 KCl, 1 CaCl_2_, 10 Glucose, 1.25 MgSO_4_, 1.25 K2HPO_4_ (pH = 7.4) for 5 minutes. The hearts were then perfused with Ca^2+^ -free Tyrode’s solution for 10 minutes followed by Ca^2+^-free Tyrode’s solution containing Collagenase B (final concentration, 0.6 mg/ml; Boehringer Mannheim, Indianapolis, IN) for an additional 6 minutes. The hearts were subsequently perfused with high-K (KB) solution containing (in mM): 70 KOH, 50 L-glutamic acid (potassium salt), 40 KCl, 10 Taurine, 2 MgCl_2_, 10 Glucose, 10 HEPES, 5 EGTA, and 1% albumin (pH 7.4, with KOH) for 5–10 minutes. The digested heart tissue was placed in fresh high-K solution, minced into smaller pieces, and triturated several times to dissociate the cells. The cell suspension was filtered through a mesh and allowed to settle for 15–20 min. Cells were stored in the KB solution at room temperature and used for patch clamp experiments on the day of isolation.

**Human-induced pluripotent stem cell** **culture.** Male human-induced pluripotent stem cell (hiPSC) line (hiPSC 522; CERVO Research Center, Québec, Canada) and female hiPSC cell line (hiPSC C1; Stanford University Cardiovascular Institute Biobank, USA) were used. The hiPSCs were cultured on hESC-qualified Matrigel (Corning, AZ, USA) in mTeSR plus medium (STEMCELL Technologies, Canada) for 5 days and differentiated into ventricular-like cardiomyocytes using the STEMdiff Ventricular differentiation kit (STEMCELL Technologies, Canada). After the period of differentiation, the media was changed every 2 days using the STEMdiff Cardiomyocyte maintenance Kit (STEMCELL Technologies). After the maturation period, we dissociated the hiPSC-derived cardiomyocytes (hiPSC-CM) monolayer into single cell at D32 using TrypLE^TM^ Express (ThermoFisher Scientific). We waited a minimum of 7 days before initiating experiments.

**Action potential recordings from guinea pig single ventricular myocyte.** Action potentials (AP) were recorded from single ventricular myocytes before and after treatment with sex hormones in the whole-cell current-clamp configuration of the patch-clamp technique using an Axopatch-200B amplifier (Axon Instruments, Inc., Burlingame, CA) by passing depolarizing currents at subthreshold intensity. To record AP, the composition of internal solution was (mM): 135 KCl , 10 EGTA, 5 Glucose, 10 HEPES, 3 Na-ATP, 0.5 Na-GTP, pH 7.3 adjusted with KOH. The external solution contained (mM): 117 NaCl, 5.7 KCl, 4.4 NaHCO_3_, 1.7 MgCl_2_, 20 HEPES, 20 Glucose, 20 Taurine, 1.8 CaCl_2_, pH 7.4 with adjusted with NaOH.

***Males.*** APs were recorded in male guinea pig myocytes, first under basal condition with external solution, and superfused during 5 minutes with the sex hormones profile observed in male controls (C, external solution with testosterone 4 ng/mL + 17-β estradiol 10 pg/mL + progesterone 0.2 ng/mL), and then sex hormones profile observed in male TdP patients (TdP, external solution with testosterone 0.1 ng/mL + 17-β estradiol 100 pg/mL + progesterone 0.2 ng/mL). Washout was performed during 5 minutes with the external solution (**Supplemental Figure 1**).

In a subsequent set of experiments, male guinea pig myocytes were superfused with the same C solution, TdP solution and followed with treatment 1 (T1, TdP solution with testosterone 4 ng/mL) and finally with treatment 2 (T2, TdP solution with testosterone 8 ng/mL) (**Supplemental Figure 2**).

***Females***. APs were recorded in female guinea pig myocytes, first under basal condition with external solution, and superfused during 5 minutes, with the sex hormones profile observed in female controls (C, external solution with testosterone 0.1 ng/mL + 17-β estradiol 10 pg/mL + progesterone 0.2 ng/mL), and then sex hormones profile observed in female TdP patients (TdP, external solution with testosterone 0.5 ng/mL + 17-β estradiol 150 pg/mL + progesterone 0.3 ng/mL). Washout was performed during 5 minutes with the external solution (**Supplemental Figure 1**).

In a subsequent set of experiments, female guinea pig myocytes were superfused with the same C solution, TdP solution and followed with treatment 1 (T1, TdP solution with progesterone 5 ng/mL) and finally with treatment 2 (T2, TdP solution with progesterone 20 ng/mL) (**Supplemental Figure 2**).

**Action potential recordings from hiPSC-CM.** APs were recorded from hiPSC-CM as we have previously described.^3^ Briefly, APs were recorded using whole cell configuration (in current clamp mode) using an AXOpatch 200B amplifier (Axon Instruments, Foster city, CA, USA). APs were elicited following injection of 3 ms, 20-1500 pA rectangular current pulse. The patch pipettes (resistance 2-5mOhm, fire-polished) were filled with a solution containing (in mmol/L): 10 NaCl, 122 KCl, 1 MgCl_2_, 1 EGTA and 10 HEPES; pH was adjusted at 7.3 with KOH. The bath solution (external current clamp) was composed of (in mmol/L): 154 NaCl, 5.6 KCl, 2 CaCl_2_, 1 MgCl_2_, 8 Glucose and 10 HEPES; the pH was adjusted at 7.3 with 1 N NaOH.

For male hiPSC-CM, APs were recorded with the bath solution (Baseline), then the sex hormones profiles observed in male controls was perfused for 5 minutes (C: testosterone 4 ng/mL + 17-β estradiol 10 pg/mL + progesterone 0.2 ng/mL). Finally, hiPSC-CMs were perfused for 5 minutes with bath solution containing the sex hormones profile observed in male Torsades de Pointes patients (TdP: testosterone 0.1 ng/mL + 17-β estradiol 100 pg/mL + progesterone 0.2 ng/mL).

For female hiPSC-CM, APs were recorded with the bath solution (Baseline), then the sex hormones profiles observed in female controls was perfused for 5 minutes (C: testosterone 0.1n g/mL + 17-β estradiol 10 pg/mL + progesterone 0.2 ng/mL). Finally, hiPSC-CMs were perfused for 5 minutes with bath solution containing the sex hormones profile observed in female Torsades de Pointes patients (TdP: testosterone 0.5 ng/mL + 17-β estradiol 150 pg/mL + progesterone 0.3 ng/mL).

APs at 90% of repolarization (APD_90_) results were measured using Clampfit (pClamp v10; Molecular devices)

**Statistical analysis**

***Handling of 17-β estradiol values below the detection limit.*** The lower detection limit for the 17-β estradiol assay was 20 pg/ml. We handled this issue in the analysis by assuming that in all cases in which the result was <20 pg/ml, the value was 10 pg/ml, i.e. the middle value between 0 and 20 pg/ml.

|  | **TdP** | **C** | ***p*** |
| --- | --- | --- | --- |
| **MALES** | | | |
| **n** | 26 | 29 |  |
| **Age, years** | 77 (66-83) | 79 (74-83) | 0.10 |
| **QTc** | 530 (520-613) | 419 (402-438) | **<0.001** |
| **Comorbidities** | | | |
| Cardiovascular disease | 20/26 (77%) | 26/29 (90%) | 0.28 |
| *Hypertension/LVH* | 9/26 (35%) | 16/29 (55%) |  |
| *CAD/vasculopathy* | 12/26 (46%) | 10/29 (34%) |  |
| *DCM/HF* | 13/26 (50%) | 6/29 (21%) |  |
| Diabetes | 11/26 (42%) | 7/29 (24%) | 0.25 |
| Chronic kidney disease | 8/26 (31%) | 5/29 (17%) | 0.34 |
| **FEMALES** | | | |
| **n** | 42 | 48 |  |
| **Age, years** | 83 (77-86) | 77.5 (71-86) | 0.30 |
| **QTc** | 600 (560-660) | 433 (420-450) | **<0.001** |
| **Comorbidities** | | | |
| Cardiovascular disease | 28/42 (67%) | 33/48 (69%) | 1.0 |
| *Hypertension/LVH* | 19/42 (45%) | 22/48 (46%) |  |
| *CAD/vasculopathy* | 8/42 (19%) | 14/48 (29%) |  |
| *DCM/HF* | 10/42 (24%) | 11/48 (23%) |  |
| Diabetes | 10/42 (24%) | 8/48 (17%) | 0.44 |
| Chronic kidney disease | 4/42 (10%) | 8/48 (17%) | 0.37 |

**Supplemental Table 1.**

**Demography and comorbidities of patients with Torsades de Pointes (TdP)**

**and controls (C) stratified by sex.**

________________________________

LVH: left ventricular hypertrophy; CAD: coronary artery disease; DCM/HF: dilated cardiomyopathy/heart failure.

Data are expressed as median (interquartile range) or frequency (percentage).

Differences in continuous variables were evaluated by the two-tail unpaired “t” test, or the two-tail Mann-Whitney test.

Difference in categorical variables were evaluated by the two-sided Fisher’s exact test.

**Supplemental Table 2.**

**Hypogonadism in male patients with Torsades de Pointes (TdP):**

**causes and laboratory findings by type and severity.**

|  | **Total** | **Peripheral** | **Central** | **Mixed** |
| --- | --- | --- | --- | --- |
| **Hypogonadism**  **(testosterone ≤2.7 ng/nl), n** | **23/26 (88%)** | 6/23 (26%) | 5/23 (22%) | 12/23 (52%) |
| Causes |  | -Androgen deprivation therapy, n=1 (bicalutamide)  -Unknown, n=5 | -Androgen deprivation therapy, n=3  (2 leuprolide,  1 triptorelin)  -Unknown, n=2 | -Active systemic inflammation, n=9  -Chronic opioid therapy,  n=3 |
| Total testosterone, ng/ml | 1.06±0.80 | 1.16±0.74 | 0.30±0.32 | 1.24±0.84 |
| Free testosterone, ng/ml | 0.016±0.015 | 0.014±0.011 | 0.003±0.004 | 0.022±0.017 |
| 17-β estradiol, pg/ml | 64.1±58.4 | 27.6±17.4 | 63.4±55.3 | 82.7±66.9 |
| LH, mU/ml | 6.03±7.19 | 16.02±7.23 | 0.18±0.24 | 3.47±1.57 |
| FSH, mU/ml | 8.93±8.78 | 20.28±10.16 | 3.40±2.00 | 5.55±3.03 |
| **Severe hypogonadism,**  **(testosterone ≤1.1 ng/nl), n** | **16/26 (62%)** | 4/16 (25%) | 5/16 (31%) | 7/16 (44%) |
| Causes |  | -Androgen deprivation therapy, n=1 (bicalutamide)  -Unknown, n=3 | -Androgen deprivation therapy, n=3  (2 leuprolide,  1 triptorelin)  -Unknown, n=2 | -Active systemic inflammation, n=6  -Chronic opioid therapy,  n=1 |
| Total testosterone, ng/ml | 0.58±0.35 | 0.71±0.33 | 0.30±0.32 | 0.64±0.35 |
| Free testosterone, ng/ml | 0.009±0.008 | 0.008±0.004 | 0.003±0.004 | 0.013±0.009 |
| 17-β estradiol, pg/ml | 75.3±63.6 | 31.5±19.5 | 63.4±55.3 | 108.9±71.9 |
| LH, mU/ml | 5.64±7.86 | 16.52±9.07 | 0.18±0.24 | 3.31±1.83 |
| FSH, mU/ml | 9.09±10.26 | 23.60±11.21 | 3.40±2.00 | 4.87±3.13 |

_______________________________

Data are expressed as mean±standard deviation or frequency (percentage).

**Supplemental Table 3.**

**Sex hormones levels in males with complicated or uncomplicated Torsades de Pointes.**

|  | **TdP**  **complicated**  **(n=14)** | **TdP**  **uncomplicated**  **(n=12)** | ***p*** |
| --- | --- | --- | --- |
| **Age**, years | 82 (52-82) | 77 (71-85) | 0.12 |
| **Total testosterone**  (r.v. 2.7-10.9 ng/ml) | 0.87±0.77 ↓ | 1.80±1.27 | **0.027*** |
| ***Subjects with low testosterone***  *(≤2.7 ng/ml), n* | 14/14 (100%) | 9/12 (75%) | 0.08 |
| ***Subjects with very low testosterone***  *(≤1.1 ng/ml), n* | 10/14 (71%) | 5/12 (42%) | 0.23 |
| **SHBG**  (r.v. 10-57 nmol/L) | 54.4±27.9 | 60.6±30.7 | 0.33 |
| **Free testosterone**  (r.v. ≥0.065 ng/ml) | 0.013±0.013 **↓** | 0.027±0.019 | **0.032*** |
| ***Subjects with very low free testosterone***  *(≤0.01 ng/ml), n* | 8/14 (57%) | 3/12 (25%) | 0.13 |
| **Androstenedione**  (r.v. 0.4-3.1 ng/ml) | 1.55±1.03 | 1.42±1.12 | 0.64 |
| **17-β estradiol**  (r.v. ≤50 pg/ml) | 66.0±55.3 | 59.4±59.1 | 0.64 |
| ***Subjects with high 17-β estradiol***  *(≥50 pg/ml), n* | 7/14 (50%) | 5/12 (42%) | 0.71 |
| ***Subjects with very high 17-β estradiol***  *(≥100 pg/ml), n* | 3/14 (23%) | 3/12 | 1.0 |
| **Progesterone**  (r.v. 0.3-0.9 ng/ml) | 0.19±0.15 | 0.27±0.30 | 0.81 |
| **LH**  (r.v. 0.8-8 mU/ml) | 3.81±3.40 | 8.39±8.87 | 0.19 |
| ***Subjects with low LH***  *(≤0.8 mU/ml), n* | 3/14 (21%) | 2/12 (17%) | 1.0 |
| ***Subjects with high LH***  *(≥8 mU/ml), n* | 2/14 (14%) | 5/12 (42%) | 0.19 |
| **FSH**  (r.v. 1.2-15.8 mU/ml) | 5.81±4.32 | 11.65±10.7 | 0.07 |
| ***Subjects with low FSH***  *(≤1.2 mU/ml), n* | 1/14 (7%) | 0/29 | 1.0 |
| ***Subjects with high FSH***  *(≥15.8 mU/ml), n* | 0/14 | 2/12 (17%) | 0.20 |

________________________________________

TdP: torsades de pointes; C: controls; SHBG: sex hormone binding globulin; LH: luteinizing hormone; FSH: follicle stimulating hormone; r.v.: reference values.

Data are expressed as mean±standard deviation or frequency (percentage).

Differences in continuous variables were evaluated by the two-tail unpaired “t” test, or the two-tail Mann-Whitney test.

Difference in categorical variables were evaluated by the two-sided Fisher’s exact test.

P values <0.05 are reported in bold and with an asterisk (*). Arrows indicate statistically significant increase/decrease in TdP patients when compared to controls.

**Supplemental Table 4.**

**Sex hormones levels in females with complicated or uncomplicated Torsades de Pointes.**

|  | **TdP**  **complicated**  **(n=22)** | **TdP**  **uncomplicated**  **(n=20)** | ***p*** |
| --- | --- | --- | --- |
| **Age**, years | 82 (76-85) | 84 (80-88) | 0.25 |
| **17-β estradiol**  (r.v. <20-60 pg/ml) | 90.7±91.7 ↑ | 41.6±41.0 | **0.034*** |
| ***Subjects with high 17-β estradiol***  *(>60 pg/ml), n* | 11/22 (50%) | 4/20 (20%) | 0.06 |
| ***Subjects with very high 17-β estradiol***  *(>100 pg/ml), n* | 8/22 (36%) ↑ | 1/20 (5%) | **0.027*** |
| **Progesterone**  (r.v. 0.1-1.1 ng/ml) | 0.28±0.31 | 0.25±0.38 | 0.67 |
| ***Subjects with high progesterone***  *(>1.1 ng/ml), n* | 1/22 (5%) | 1/20 (5%) | 1.0 |
| **Total testosterone**  (r.v. 0.1-1.1 ng/ml) | 0.49±0.57 | 0.29±0.25 | 0.19 |
| ***Subjects with high testosterone***  *(>1.1 ng/ml), n* | 1/22 (5%) | 0/20 | 1.0 |
| **SHBG**  (r.v. 18-144 nmol/L) | 62.5±46.7 | 63.6±37.8 | 0.60 |
| **Free testosterone**  (r.v. <0.01 ng/ml) | 0.007±0.010 | 0.017±0.065 | 0.14 |
| ***Subjects with high free testosterone***  *(>0.01 ng/ml), n* | 4/22 (18%) | 0/20 | 0.11 |
| ***Subjects with very high free***  ***testosterone***  *(>0.065 ng/ml), n* | 0/22 | 0/49 | 1.0 |
| **Androstenedione**  (r.v. 0.4-4.1 ng/ml) | 2.46±2.30 ↑ | 1.42±1.31 | **0.038*** |
| **LH**  (r.v. 11-40 mU/ml) | 8.9±6.6 | 11.7±9.1 | 0.25 |
| ***Subjects with low LH***  *(<11 mU/ml), n* | 14/22 (64%) | 11/20 (55%) | 0.75 |
| **FSH**  (r.v. 35-160 mU/ml) | 22.5±17.3 | 32.3±22.1 | 0.11 |
| ***Subjects with low FSH***  *(<35 mU/ml), n* | 16/22 (73%) | 11/20 (55%) | 0.34 |

***_________________________________***

TdP: torsades de pointes; C: controls; SHBG: sex hormone binding globulin; LH: luteinizing hormone; FSH: follicle stimulating hormone; r.v.: reference values.

Differences in continuous variables were evaluated by the two-tail unpaired “t” test, or the two-tail Mann-Whitney test.

Difference in categorical variables were evaluated by the two-sided Fisher’s exact test.

P values <0.05 are reported in bold and with an asterisk (*). Arrows indicate statistically significant increase/decrease in TdP patients when compared to controls.

**Supplemental Table 5.**

**Sex hormones concentrations used in *in-vitro* electrophysiological experiments, and relationship with circulating levels observed in TdP patients and controls.**

| **Hormone** | **Males** | | **Females** | |
| --- | --- | --- | --- | --- |
|  | Control | TdP | Control | TdP |
| **Testosterone**  (ng/ml) | **4**  (*Q3*: 3.84) | **0.1**  (*Q1*: 0.14)* | **0.1**  (*Q1*: 0.08) | **0.5**  (*Q3*: 0.54)* |
| **17β-estradiol** (pg/ml) | **10**  (*Q1*: 10.0) | **100**  (*Q3*: 96.2)* | **10**  (*Q1*: 10.0) | **150**  (*Q3*: 146.0)* |
| **Progesterone** (ng/ml) | **0.2**  (*mean*: 0.23) | **0.2**  (*mean*: 0.16) | **0.2**  (*Q1*: 0.16) | **0.3**  (*Q3*: 0.30)* |

***_________________________________***

TdP: torsades de pointes; Q1: first quartile; Q3: third quartile.

*Values observed in complicated TdP patients.


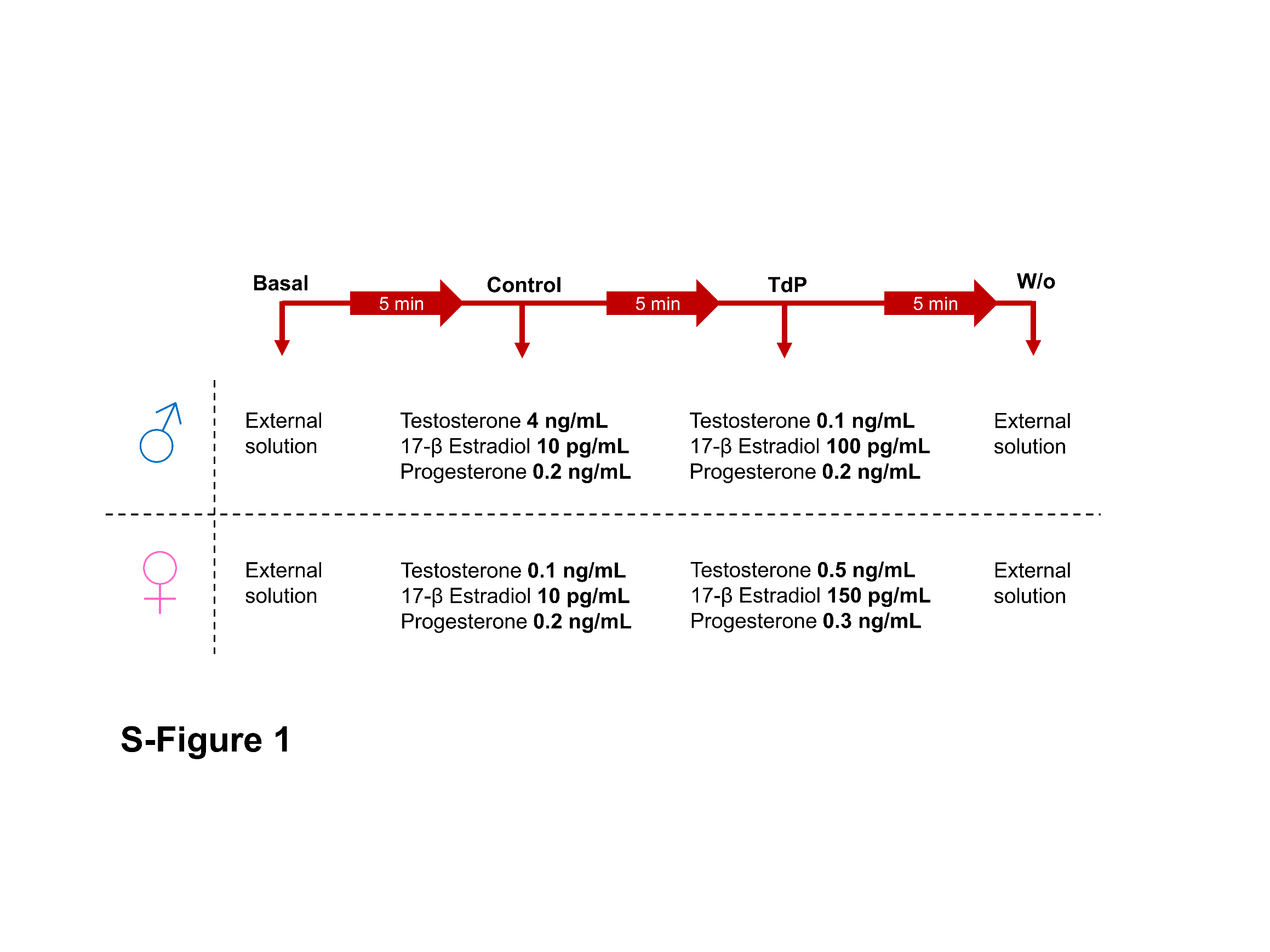


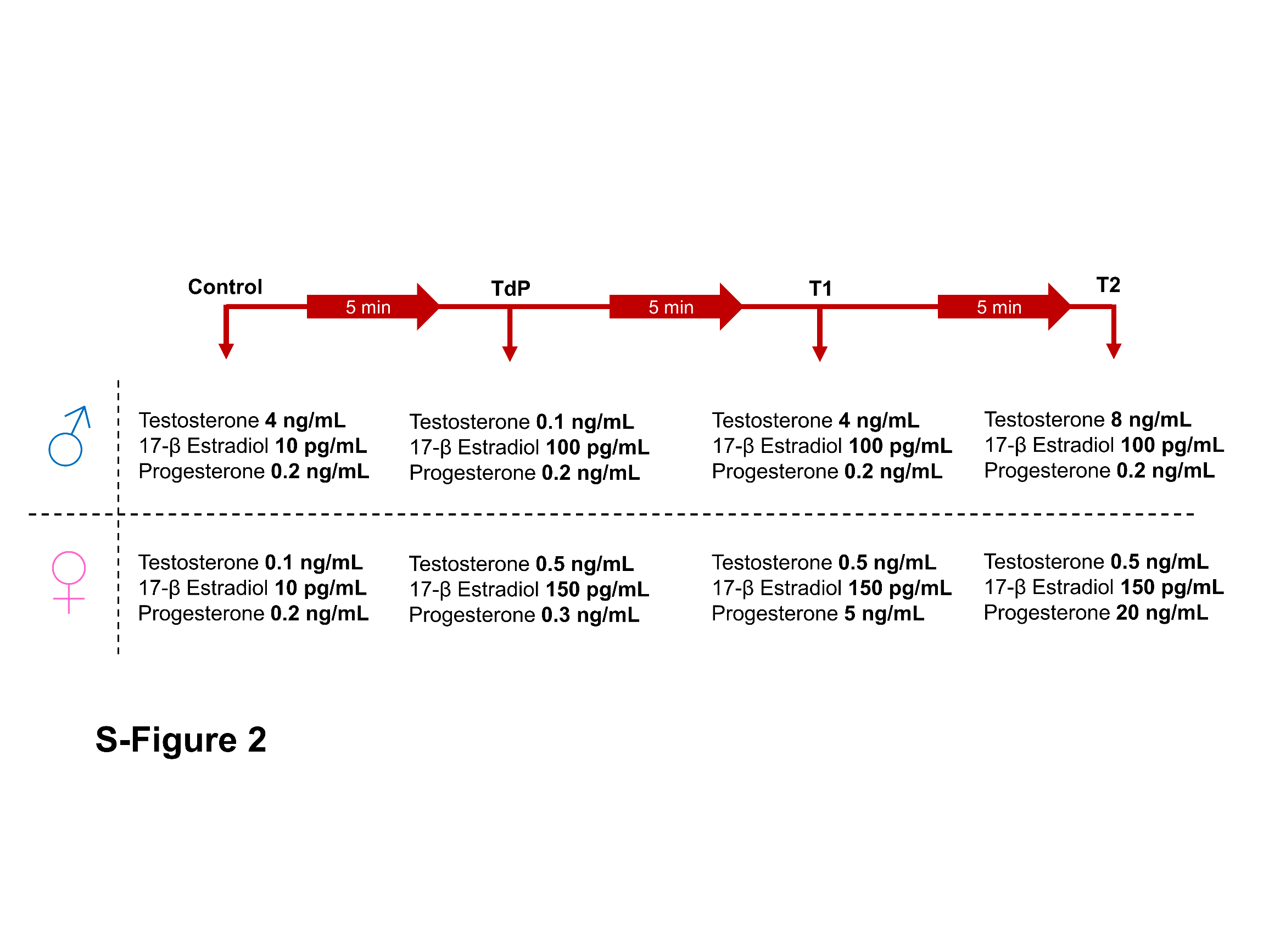


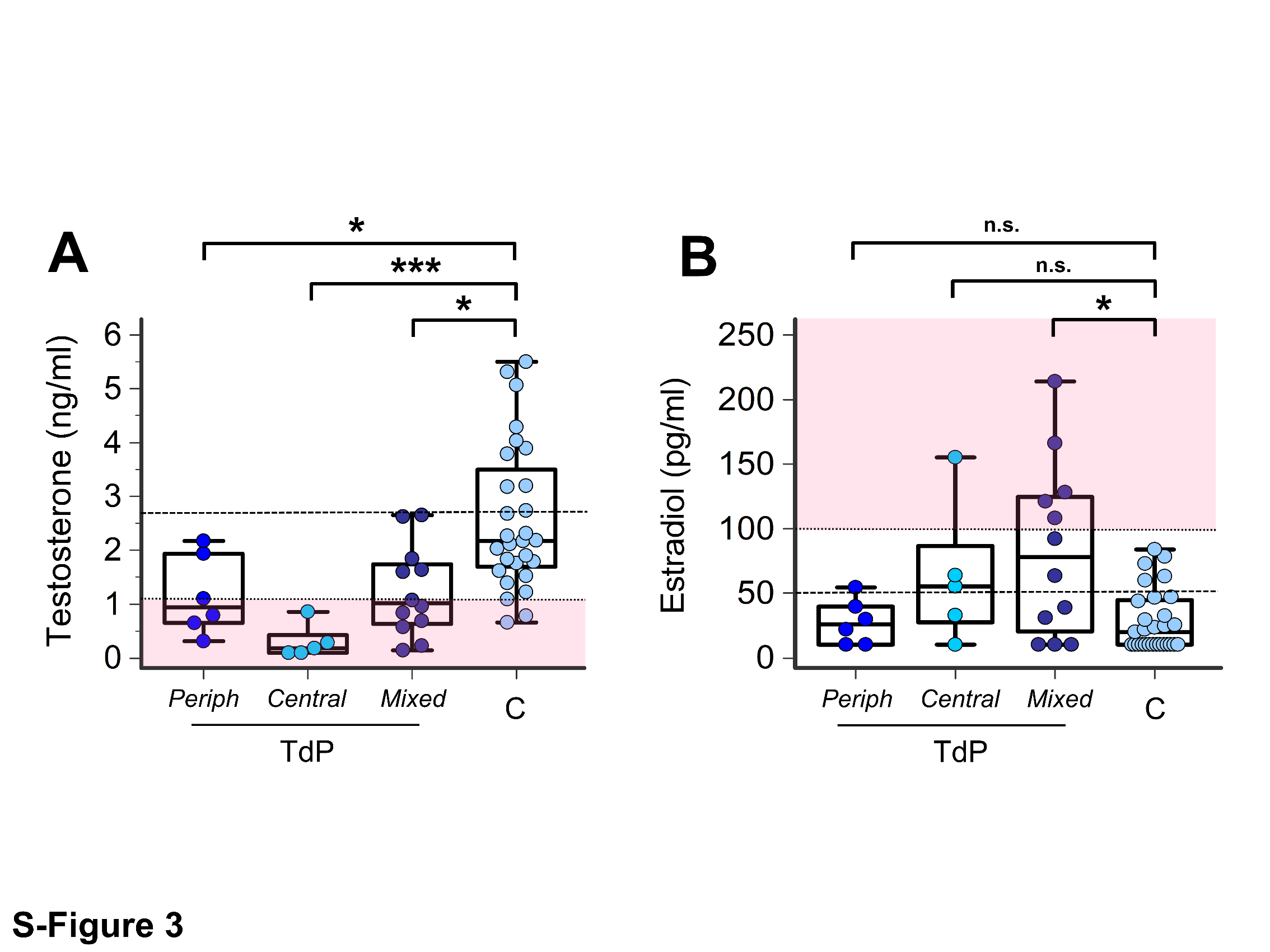


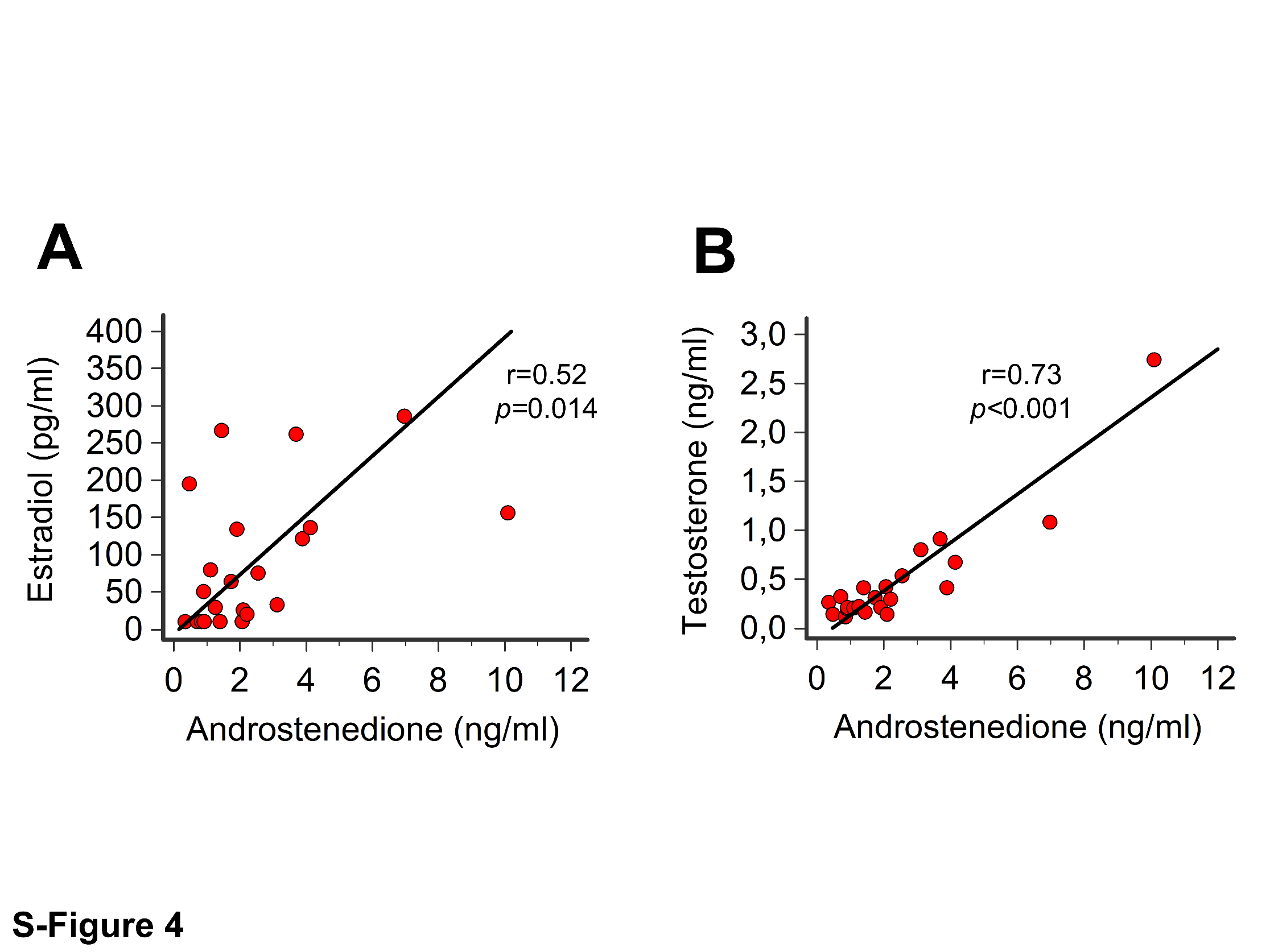


**
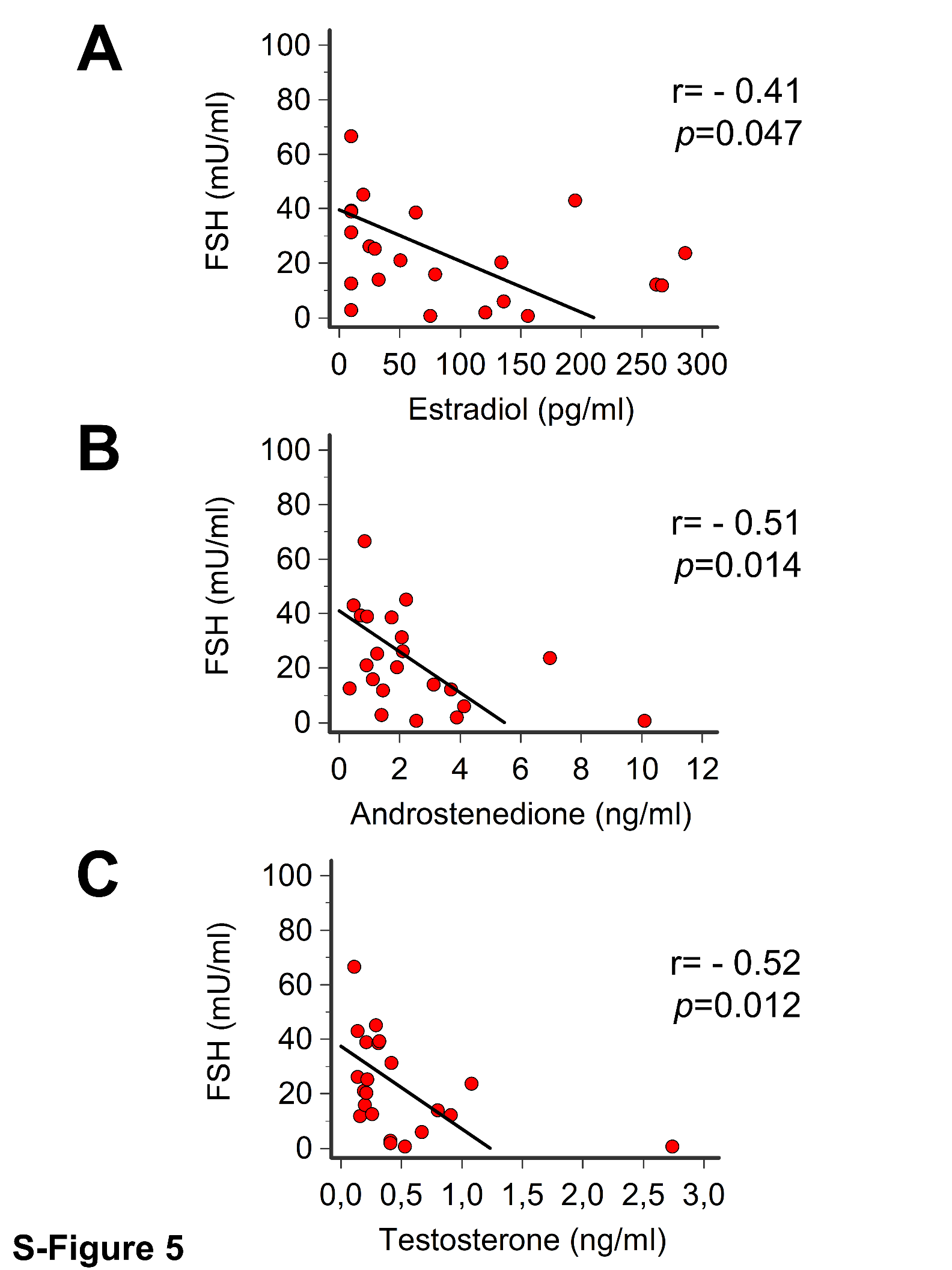
**

**
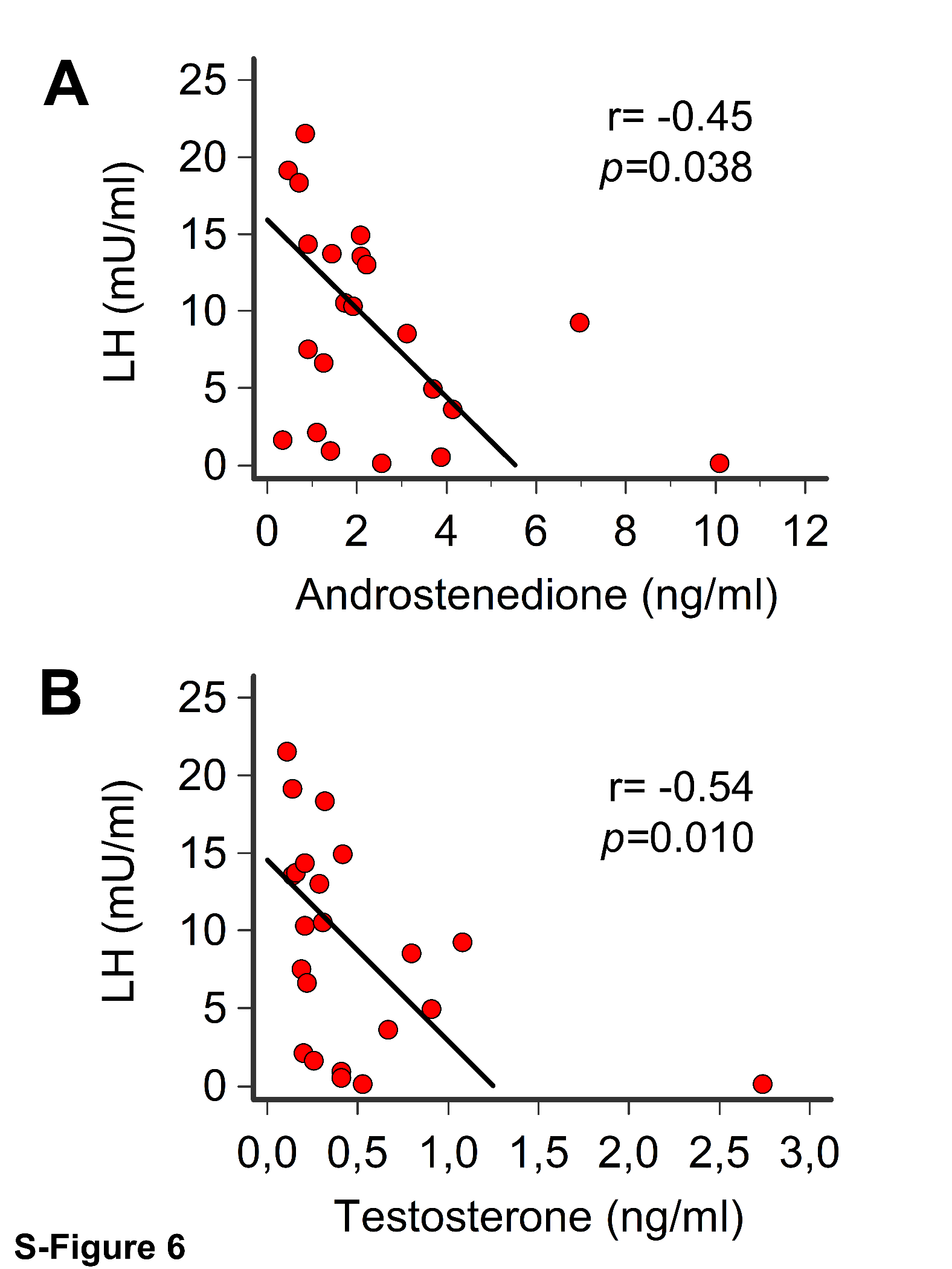
**

**S-Figure 7**

**
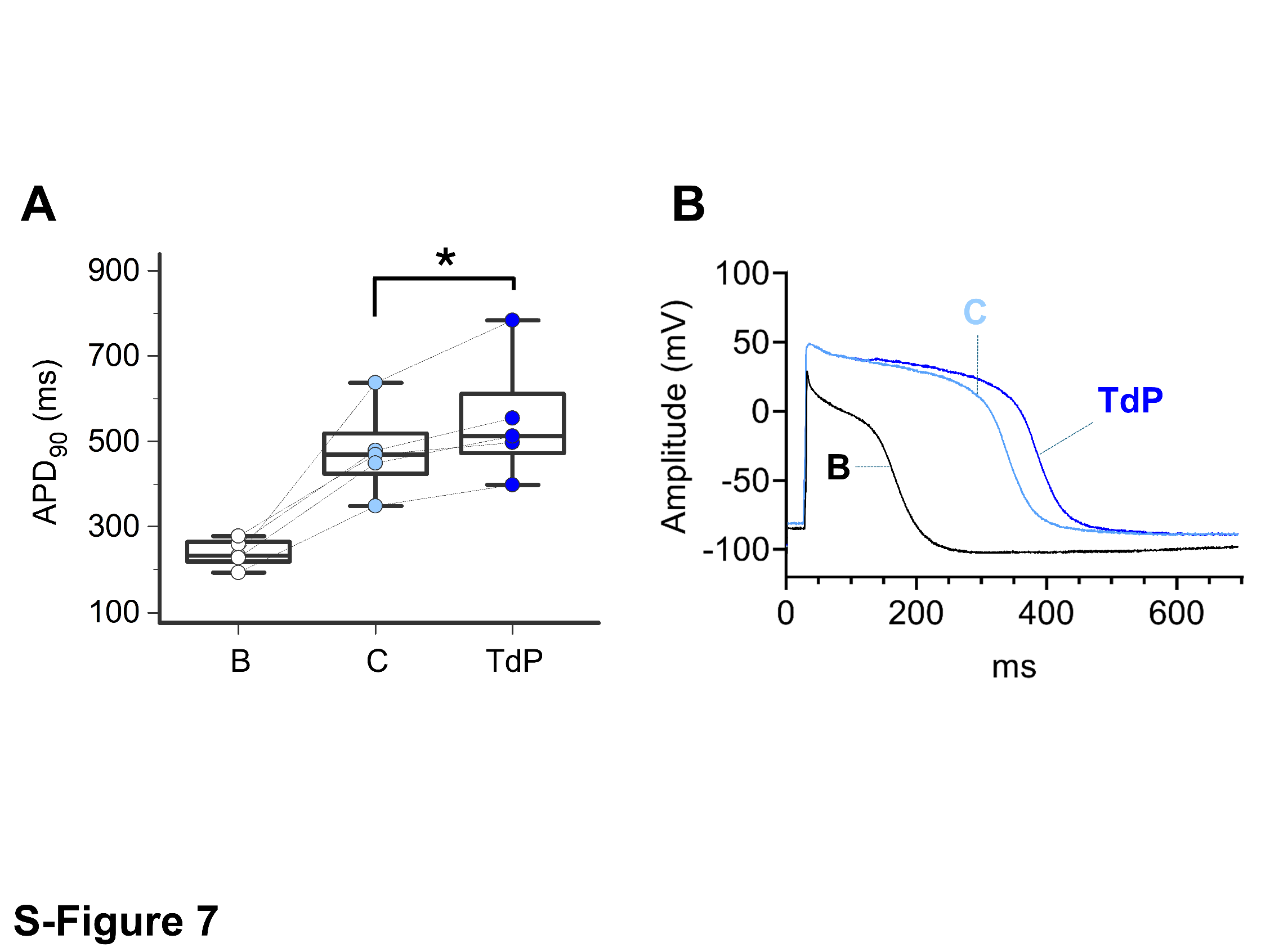
**


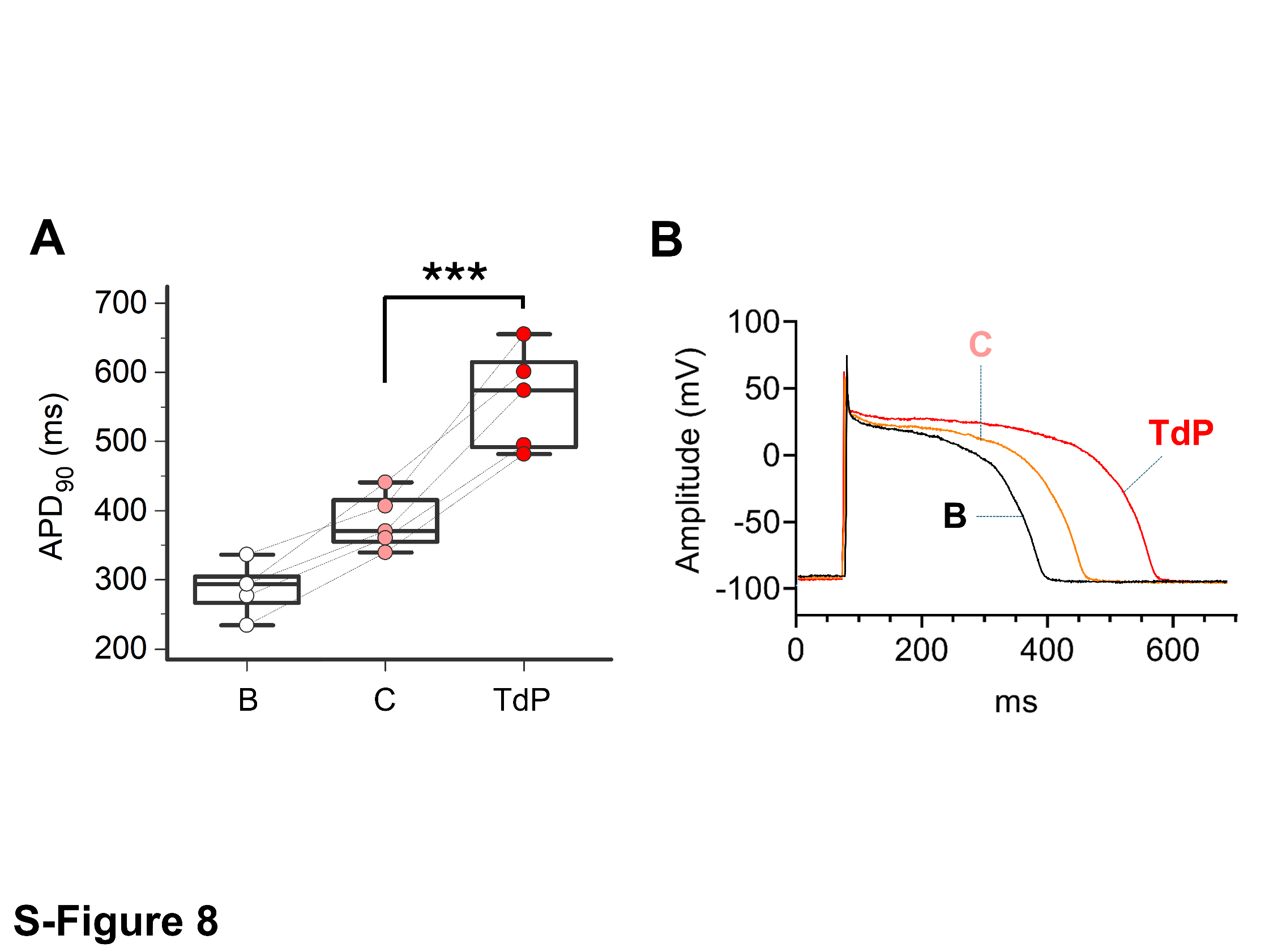


**S-Figure 8**

**LEGENDS TO SUPPLEMENTAL FIGURES**

**Supplemental Figure 1. Experimental protocol used to evaluate the** ***in-vitro* impact of sex hormones profiles observed in male and female TdP patients on guinea-pig ventricular myocyte APD**.

APD: action potential duration; TdP: Torsades de Pointes; W/o: washout.

**Supplemental Figure 2. Experimental protocol used to evaluate the** ***in-vitro* effect of sex-specific hormonal interventions in reverting TdP profile-associated guinea-pig ventricular myocyte APD prolongation.**

APD: action potential duration; TdP: Torsades de Pointes; T1: treatment 1; T2: treatment 2.

**Supplemental Figure 3. Circulating testosterone and 17-β estradiol levels in hypogonadic males with Torsades de pointes (TdP) stratified for hypogonadism type (peripheral, central, mixed), and controls (C).** TdP patients, n=23; C, n=29. (**A**) Total testosterone. Horizontal black dotted line indicates the lower limit of reference values in men, i.e. 2.7 ng/ml; horizontal dotted line with pink below indicates the upper limit of reference values in premenopausal women, i.e. 1.1 ng/ml. Kruskal-Wallis test, p<0.001; Dunn post-hoc multiple comparison test, *p<0.05, ***p<0.001. (**B**) 17-β estradiol. Horizontal black dotted line indicates the upper limit of reference values in men, i.e. 50 pg/ml; horizontal dotted line with pink above indicates values included in a premenopausal female range, i.e. ≥100 pg/ml. Kruskal-Wallis test, p=0.031; Dunn post-hoc multiple comparison test, *p<0.05.

Estradiol: 17-β estradiol.

**Supplemental Figure 4. Correlation between 17-β estradiol, testosterone, and androstenedione levels in female patients with complicated Torsades de Pointes.** **A**. Relationship between 17-β estradiol and androstenedione levels. **B**. Relationship between testosterone and androstenedione levels.

Spearman test. Patients, n=22. Estradiol: 17-β estradiol.

**Supplemental Figure 5. Correlation between 17-β estradiol, androstenedione, testosterone, and FSH levels in female patients with complicated Torsades de Pointes.** **A**. Relationship between 17-β estradiol and FSH levels. **B**. Relationship between androstenedione and FSH levels. **C**. Relationship between testosterone and FSH levels.

Spearman test. Patients, n=22. FSH: follicle stimulating hormone; estradiol: 17-β estradiol.

**Supplemental Figure 6. Correlation between androstenedione, testosterone, and LH levels in female patients with complicated Torsades de Pointes.** **A**. Relationship between androstenedione and LH levels. **C**. Relationship between testosterone and LH levels.

Spearman test. Patients, n=22. LH: luteinizing hormone.

**Supplemental Figure 7. In-vitro impact of sex hormones profiles observed in male patients with Torsades de Pointes (TdP) and controls (C) on human induced pluripotent stem cells (hiPSCs) derived cardiomyocytes action potential duration (APD). (A)** Action potential duration at 90% (APD 90) measured in hiPSC derived cardiomyocytes (n=5 cells) perfused with regular Tyrode solution (Baseline, B), the sex hormones profile observed in male controls (C: testosterone 4 ng/mL + 17-β estradiol 10 pg/mL + progesterone 0.2 ng/mL), and the sex hormones profile observed in male Torsades de Pointes patients (TdP: testosterone 0.1 ng/mL + 17-β estradiol 100 pg/mL + progesterone 0.2 ng/mL). **(B)** Representative traces of hiPSC derived cardiomyocytes APD90 after different treatments. Repeated measures analysis of variance (RM-ANOVA), p=0.007; post-hoc multiple paired t test with false discovery rate (fdr) correction: *p<0.05.

**Supplemental Figure 8. In-vitro impact of sex hormones profiles observed in female patients with Torsades de Pointes (TdP) and controls (C) on human induced pluripotent stem cells (hiPSCs) derived cardiomyocytes action potential duration (APD). (A)** Action potential duration at 90% (APD90) measured in hiPSC derived cardiomyocytes (n=5 cells) perfused with regular Tyrode solution (Baseline, B), the sex hormones profile observed in female controls (C: testosterone 0.1 ng/mL + 17-β estradiol 10 pg/mL + progesterone 0.2 ng/mL), and the sex hormones profile observed in female Torsades de Pointes patients (TdP: testosterone 0.5 ng/mL + 17-β estradiol 150 pg/mL + progesterone 0.3 ng/mL). **(B)** Representative traces of hiPSC derived cardiomyocytes APD90 after different treatments. Repeated measures analysis of variance (RM-ANOVA), p<0.001; post-hoc multiple paired t test with false discovery rate (fdr) correction: ***p<0.001.

**Supplemental References**

1. Gupta A, Lawrence AT, Krishnan K, Kavinsky CJ, Trohman RG. Current concepts in the mechanisms and management of drug-induced QT prolongation and torsade de pointes. *Am Heart J*. 2007;153:891-899. doi: 10.1016/j.ahj.2007.01.040

2. Drew BJ, Ackerman MJ, Funk M, Gibler WB, Kligfield P, Menon V, Philippides GJ, Roden DM, Zareba W, American Heart Association Acute Cardiac Care Committee of the Council on Clinical Cardiology tCoCN, and the American College of Cardiology Foundation. Prevention of torsade de pointes in hospital settings: a scientific statement from the American Heart Association and the American College of Cardiology Foundation. *Circulation*. 2010;121:1047-1060. doi: 10.1161/CIRCULATIONAHA.109.192704

3. Reisqs JB, Moreau A, Sleiman Y, Charrabi A, Delinière A, Bessière F, Gardey K, Richard S, Chevalier P. Spironolactone as a Potential New Treatment to Prevent Arrhythmias in Arrhythmogenic Cardiomyopathy Cell Model. *J Pers Med*. 2023;13. doi: 10.3390/jpm13020335
